# Cellulose-binding activity of a 21-kDa endo-ß-1,4-glucanase lacking cellulose-binding domain and its synergy with other cellulases in the digestive fluid of *Aplysia kurodai*

**DOI:** 10.1371/journal.pone.0205915

**Published:** 2018-11-09

**Authors:** Akihiko Tsuji, Keizo Yuasa, Chikako Asada

**Affiliations:** 1 Department of Biomolecular Function and Technology, Graduate School of Bioscience & Bioindustry, Tokushima University, Minamijosanjima, Tokushima, Japan; 2 Department of Bioresource Chemistry and Technology, Graduate School of Bioscience & Bioindustry, Tokushima University, Minamijosanjima, Tokushima, Japan; University of Tennessee, UNITED STATES

## Abstract

Endo-ß-1,4-glucanase AkEG21 belonging to glycosyl hydrolase family 45 (GHF45) is the most abundant cellulase in the digestive fluid of sea hare (*Aplysia kurodai*). The specific activity of this 21-kDa enzyme is considerably lower than those of other endo ß-1,4-glucanases in the digestive fluid of *A*. *kurodai*, therefore its role in whole cellulose hydrolysis by sea hare is still uncertain. Although AkEG21 has a catalytic domain without a cellulose binding domain, it demonstrated stable binding to cellulose fibers, similar to that of fungal cellobiohydrolase (CBH) 1 and CBH 2, which is strongly inhibited by cellohexaose, suggesting the involvement of the catalytic site in cellulose binding. Cellulose-bound AkEG21 hydrolyzed cellulose to cellobiose, cellotriose and cellotetraose, but could not digest an external substrate, azo-carboxymethyl cellulose. Cellulose hydrolysis was considerably stimulated by the synergistic action of cellulose-bound AkEG21 and AkEG45, another ß-1,4-endoglucanase present in the digestive fluid of sea hare; however no synergy in carboxymethylcellulose hydrolysis was observed. When AkEG21 was removed from the digestive fluid by immunoprecipitation, the cellulose hydrolyzing activity of the fluid was significantly reduced, indicating a critical role of AkEG21 in cellulose hydrolysis by *A*. *kurodai*. These findings suggest that AkEG21 is a processive endoglucanase functionally equivalent to the CBH, which provides a CBH-independent mechanism for the mollusk to digest seaweed cellulose to glucose.

## Introduction

Plant cellulose presents the most abundant renewable biomass on Earth and the conversion of lignocellulose to biofuel by cellulolytic enzymes is considered a promising approach for providing renewable energy. The filamentous fungus *Trichoderma reesei* (telemorph *Hypocrea jecorina*) is widely used as a source of cellulolytic enzymes in bioindustry, mainly in the production of biofuels since it produces a variety of enzymes in large quantities, including ß-1,4-endoglucanases, cellobiohydrolases (CBHs) and ß-glucosidases [1, 2]. In *Trichoderma* species, an individual enzyme alone cannot completely depolymerize crystalline cellulose, but the synergistic action of the enzyme mixture provides efficient saccharification of plant cell wall insoluble cellulose [3]. *T*. *reesei* secretes different types of cellulases possessing distinct cleavage specificity toward cellulose and oligocellulose including cellobiohydrolases, Cel7A (CBH 1) and Cel6A (CBH 2), endoglucanases (e.g., Cel 6B and Cel7B), and ß-glucosidases. CBH1, CBH2 and Cel7B are the three major components of the *T*. *reesei* cellulase system, the former two accounting for 60% and 20%, respectively, of total cellulase proteins [4–6]. CBHs play a critical role in the saccharification of crystalline cellulose. *T*. *reesei* CBHs Cel7A (CBH 1) and Cel6A (CBH 2) are processive cellulases acting on crystalline cellulose from reducing and non-reducing ends, respectively [7–9]. However, in contrast to *T*. *reesei*, some fungi do not express large amounts of CBHs [10]. For example, the brown-rot fungus *Postia placenta* can decompose wooden structures and possesses strong cellulose hydrolytic activity despite the absence of CBH-encoding genes [11, 12], suggesting that CBH functions are substituted by the activity of unique endoglucanases [13, 14]. A CBH-independent mechanism was also reported for a marine bacterium (*Saccharophagus degradans*) [15].

Generally, most endo-ß-1,4-glucanases and CBHs contain a catalytic domain connected to one or more non-catalytic cellulose binding domains (CBDs) through a linear linker with a flexible secondary structure offering sufficient space between the two domains [16, 17]. By binding to cellulose, the CBD increases the effective enzyme concentration on the surface of cellulose fibers, thus promoting hydrolysis of the insoluble substrate and disruption of the crystalline cellulose structure [16]. Furthermore, non-catalytic cellulose binding proteins enhancing enzymatic cellulose digestion have also been identified [18, 19]. Expansins, which have sequence homology to cellulase catalytic domains of glycosyl hydrolase family 45 (GHF 45) enzymes, bind to cellulose and induce extension and relaxation of the plant cell wall, resulting in loosening of the rigid ligocellulostic matrix and acceleration of cellulose hydrolysis by cellulolytic enzymes. Cellulose-binding expansins and expansin-like proteins have been found in plants and microorganisms including *T*. *reesei* in which swollenin, an expansin-like protein has been identified [19]. Thus, CBD-containing cellulases and non-catalytic cellulose binding proteins play an important role in cellulose hydrolysis in plants, fungi and bacteria.

On the other hand, there is increasing interest in algae as one of the alternative renewable sources of biofuels and biochemicals [20, 21]. *Aplysia kurodai* thrives on seaweed and presents a good source of cellulose hydrolases as its gastric system produces considerable volumes of the digestive fluid. Recently we investigated the entire enzymatic cellulolytic system of the digestive fluid from *A*. *kurodai* with the aim to provide useful clues for the establishment of an effective enzymatic saccharification of seaweeds [22]. As a result, we identified four endo-ß-1,4-glucanases (with molecular masses of 95, 65, 45 and 21 kDa) and two ß-glucosidases (210 and 110 kDa) and analyzed their synergistic activity in cellulose digestion. Sequence analysis indicated that the 21 kDa cellulase (AkEG21) belongs to GHF45, whereas the 45, 65 and 95 kDa cellulases belong to GHF9 [22, 23]. To date, it has been shown that AkEG21 dose not possess a cellulose binding domain [23] similar to the GHF45 endoglucanase from the insect *Apriona japonica* [24].

Similar to the microbial cellulose-degrading system, cellulose-binding activities of cellulases and non-catalytic cellulose-binding proteins of *A*. *kurodai* are presumed to play an important role in cellulose digestion. In this study, we aimed to identify cellulose-binding proteins in the digestive fluid of *A*. *kurodai*. Surprisingly, we found that CBD-lacking AkEG21 (21 kDa endo-ß-1,4-glucanase) was a major cellulose-binding protein and characterized its cellulose-binding activity and role in cellulose digestion by sea hare. AkEG21 showed synergistic activity with other *A*. *kurodai* cellulases and cellulose hydrolysis by the digestive fluid was markedly decreased by the removal of AkEG21 through immunoprecipiation. Our results revealed a significant role of GHF45 cellulase, AkEG21 in seaweed cellulose digestion by sea hare.

## Materials and methods

### Materials

*A*. *kurodai* (body length, 20–25 cm) was collected during April—July on the coast of Naruto, Japan, where sea hare is not protected and no specific permissions is required for the collection. The field studies did not involve endangered or protected species. The digestive fluid was obtained from the gastric lumen by squeezing the stomach after dissection and stored at -30°C until use.

Carboxymethylcellulose (CMC, sodium salt, low viscosity), Avicel (Avicel PH-101) and cellulose fiber (CF-11) were purchased from Sigma-Aldrich (St, Louis, MO, USA). Filter paper (3MM) was from GE Healthcare (Buckinghamshire, U.K.). Bemcot, a wiper made from Bemliese (cupro continuous filament non-woven product made from cotton linter) was from Asahi-Kasei (Tokyo, Japan). Bemcot has a crystal structure of cellulose II and its crystallinity is three times lower than that of microcrystalline cellulose [25]. Cellotriose, cellotetraose, cellopentaose and cellohexaose were from Carbosynth (Berkshire, UK). Curdlan, starch, soluble starch (starch partial digest), chitin, Glucose CII Test Wako Kit and silver stain kit Wako were from Wako Pure Chemicals (Osaka, Japan). Azo-CMC was from Megazyme (Bray, Ireland). DEAE-Sepharose^TM^ (fast flow), CM-Sepharose^TM^ (fast flow), phenyl-Sepharose (HiLoad^TM^ 16/10), Sephacryl S-100 and Sephacryl S-200 were obtained from GE Healthcare (Uppsala, Sweden). Meicelase (lyophilized powder of cellulases secreted by *Trichoderma viride*) was purchased from Meiji-Seika (Tokyo, Japan). All other chemicals used were of analytical grade.

### Enzyme assay

Cellulase activity was assessed using CMC as a substrate according to a previously described protocol [26]. The amount of reducing sugar liberated by the hydrolysis of the substrate was determined by the method of Nelson and Somogyi [27]. Cellulolytic activity toward CF-11, Avicel, filter paper and Bemcot was determined as follows. Ten mg of CF-11, Avicel, filter paper or Bemcot was incubated with the enzyme in a 1.0 mL of 50 mM sodium acetate buffer (pH 5.5). All reactions were performed at pH 5.5 unless otherwise stated because the pH of the digestive fluid of sea hare is pH 5.5 [22]. After the reactions were complete, 100 μL of reaction mixture was lyophilized, dissolved with 10 μL of distilled water, and 1.0 μL was used for analysis by thin layer chromatography (TLC). TLC was performed using TLC Silica gel 60F plates (Merck KGaA, Darmstadt, Germany) and a developing solvent comprised of 1-buthanol/acetic acid/water (2:1:1, v/v/v). The products developed on the plate were detected using orcinol-sulfuric acid [28]. The amount of glucose released through substrate hydrolysis was determined by the Glucose CII Test Wako Kit using glucose oxidase.

Cellulase activity of cellulose-bound AkEG21 was determined by incubating 10 mg of Bemcot, CF-11, Avicel or filter paper with 50 μg of AkEG21 in 1.0 mL of 50 mM sodium acetate buffer (pH 5.5) at 4°C for 1 h, and further incubating the reaction in buffers of different pHs and /or for different periods of time. The reaction products were then analyzed by TLC as mentined above. The amount of total hexose liberated by the hydrolysis of cellulose was determined by the phenol-sulfuric acid method [29]. The activity toward an external substrate was determined as follows: AkEG21 (0.1 mg) was incubated with 100 mg of filter paper (20 pieces of 0.5 cm x 0.5 cm) in 1.0 mL of 50 mM sodium acetate buffer (pH 5.5) at room temperature for 2 h. The supernatant and AkEG21-bound filter paper were separated by centrifugation. The filter paper was washed with 1.0 mL of 50 mM sodium acetate buffer (pH 6.0) three times by centrifugation. AkEG21 (0.1 mg) incubated without filter paper was used as a control. The cellulase activity toward azo-CMC of the supernatant incubated in the absence or presence of filter paper was assayed using a 0.1 mL aliquot (1/10 of the total amount) according to the manufacturer’s protocol. The activity of AkEG21 bound to filter paper was assayed using four pieces of paper filter (20 mg, 1/5 of the total amount). Relative enzyme activity (mean ± standard deviation) was calculated from three separate determinations. ß-Glucosidase (BGL) activity was assayed using 4-methylumbelliferyl (4MU)-ß-glucoside, as described previously [26]. Protein concentration was determined by the method of Bradford using BSA as a standard [30].

### Purification of AkEG21, AkEG45 and 110K BGL from digestive fluid

AkEG21, AkEG45 (45 kDa ß-1,4-endoglucanase) and 110 K BGL (110 kDa ß-glucosidase) were purified from the digestive fluid of *A*. *kurodai* by ammonium sulfate fractionation (30–60% saturation) and chromatographies using CM-Sepharose, DEAE-Sepharose, phenyl-Sepharose and Sephacryl S-100 as described previously [22].

### Purification of CBH 1 and 2 from Meicelase

Meicelase (200 mg of lyophilized powder) was dissolved in 4.0 mL of 20 mM sodium acetate buffer (pH 6.0) containing 0.1 M NaCl, applied to a Sephacryl S-200 column (2.5 x 96 cm) and eluted with the same buffer. The fractions possessing ß-glucosidase activity were concentrated, dialyzed against 20 mM sodium acetate buffer (pH 6.0) and loaded into a DEAE-Sepharose column (1.0 x 8.0 cm) equilibrated with the same buffer. BGL1 (Cel 3A) was found in the passed through fraction and the bound proteins were eluted by a linear gradient of NaCl (0–0.2 M). CBH 2, EG-II and CBH 1 were eluted as separated single peaks. Since the amino-terminus of CBH 1 and CBH 2 were blocked, these proteins were digested with trypsin and identified by internal peptide sequencing.

### Sequence analysis

The purified enzyme or trypsin-digested enzyme fragments separated by SDS-PAGE [31] were electrotransferred onto a PVDF membrane (Immobilion^TM^, 0.45 μm, Millipore, Bedford, MA, USA) according to the manufacturer’s instruction. The protein band stained with Ponceau S was applied to an automated protein sequencer (Shimadzu PPSQ-10, Kyoto, Japan) and the N-terminal sequence was determined. For internal sequence analysis, the protein band was digested with lysyl endopeptidase [32], the released peptide was purified by reversed-phase high-performance liquid chromatography and amino acid sequences were analyzed using an automated protein sequencer (PPSQ-10, Shimadzu, Kyoto, Japan).

### Screening of cellulose binding proteins in the digestive fluid of sea hare

Digestive fluid (0.4 mL) was incubated with 10 mg of CF-11 or starch suspended in 0.4 mL of 50 mM sodium acetate buffer (pH 5.5) at 4°C with continuous rotation for 2 h. Starch was used without heat treatment. The reaction mixtures were centrifuged at 12,000 x g for 1 min. Precipitates were washed four times with 1.0 mL of 20 mM sodium acetate buffer (pH 6.0) containing 0.1 M NaCl by centrifugation, suspended in 0.5 mL of 1.0% SDS, 10 mM Tris-HCl (pH 7.0) and incubated at 37°C for 1 h. After centrifugation, the supernatant was lyophilized, dissolved in 0.1 mL of the loading buffer containing ß-mercaptoethanol, denatured at 95°C for 5 min and 10 μL was applied to a 12% SDS-PAGE gel. The aliquot for SDS-PAGE was treated with the loading buffer containing ß-mercaptoethanol according to Laemmli’s method [31] unless otherwise stated. Protein bands were detected by Coomassie Brilliant Blue (CBB) or Ponceau S staining and subjected to amino acid sequencing.

### Screening of cellulose-binding proteins in Meicelase

Meicelase (10 mg) was incubated with 20 mg of CF-11 or starch in 0.5 mL of 50 mM sodium acetate buffer (pH 5.5) at 4°C with continuous rotation for 16 h. The reaction mixture was centrifuged and precipitate was washed five times with 1.0 mL of 50 mM acetate buffer (pH 6.0) containing 0.1 M NaCl and bound proteins were analyzed as described above. For the enzymatic characterization of proteins bound to cellulose, Meicelase (4.0 mL, 3.1 mg protein/mL) was incubated with 200 mg of Avicel or Bemcot at 37°C for 30 min and centrifuged at 12,000 x g for 10 min. The resulting supernatants were incubated with 200 mg of Avicel or Bemcot again at 37°C for 30 min and centrifuged. The first Avicel incubated with Meicelase was washed five times with 10 mL of 20 mM sodium acetate buffer (pH 6.0) containing 0.1 M NaCl. To identify enzymatic activity of proteins bound to the Avicel, washed Avicel (0.1 mL) was incubated in 0.4 mL of 50 mM sodium acetate buffer (pH 5.5) at 37°C for 0–2 h and reaction products liberated from Avicel were analyzed by TLC and orcinol-sulfuric acid staining [28] as described above. The supernatants were dialyzed against 20 mM sodium acetate buffer (pH 6.0) to remove reaction products released from Avicel or Bemcot during incubation with Meicelase and used for comparison of protein compositions and glucose-producing activity from the filter paper with those of the control Meicelase. The dialysate (0.1 mL) was incubated with 10 mg of filter paper in 50 mM sodium acetate buffer (pH 5.5) at 37°C for 1 h and glucose liberated from the filter paper was determined using Glucose CII Test Wako Kit.

### Analysis of AkEG21 binding specificity

Purified AkEG21 (20 μg) was incubated with 10 mg of filter paper, CF-11, Avicel, curdlan, starch or chitin in 1.0 mL of 50 mM sodium acetate buffer (pH 5.5) at 4°C for 16 h. All polysaccharides were dissolved or suspended in the buffer without heat treatment and used for the binding experiments. The reaction mixtures were centrifuged and precipitates were washed three times with 1.0 mL of 20 mM sodium acetate buffer (pH 6.0) containing 0.1 M NaCl by centrifugation, dissolved in 0.2 mL of the loading buffer in the presence and absence of ß-mercatoethanol and incubated at 37°C for 30 min. Then a 20 μL aliquots was analyzed by SDS-PAGE in a 12% gel.

AkEG21 binding to CMC was analyzed using gel filtration on Sephacryl S-100. Purified AkEG21 (1 mg) was incubated with 3.0 mL of 1% CMC in 50 mM sodium acetate buffer (pH 5.5) at 37°C for 30 min or 24 h, and applied to a Sephacryl S-100 column (2.0 x 110 cm) equilibrated with 20 mM sodium acetate buffer (pH 6.0) containing 0.1 M NaCl. CMC digestion products and AkEG21 were eluted from the column using 20 mM sodium acetate buffer (pH 6.0) containing 0.1 M NaCl at a flow rate of 20 mL/h and 2.6 mL fractions were collected. The amount of total hexose in fractions was determined by the phenol-sulfuric acid method [26]. Elution profile of AkEG21 was determined by SDS-PAGE under reducing condition. The eluate (20 μL) was applied to 12% SDS-PAGE and protein was detected by silver staining using silver stain kit Wako. As controls, 1% CMC (3 mL) alone and AkEG21 (1 mg) incubated at 37°C for 30 min and 24 h in the absence of CMC were also analyzed.

### Effect of different reaction conditions on AkEG21 binding to cellulose

AkEG21 (20 μg) was incubated with 10 mg of CF-11 and Bemcot in 0.5 mL of 50 mM sodium acetate buffer (pH 4.0, 5.5, 6.0, 6.5) or Tris-HCl buffer (pH 7.0 and 8.0) at 4°C for 16 h. After washing five times with 0.5 mL of 50 mM sodium acetate buffer (pH 5.5), CF-11 and Bemcot were incubated in 0.2 mL of the loading buffer containing ß-mercaptoethanol at 95°C for 5 min and analyzed by SDS-PAGE.

To determine effects of ionic strength, detergents and alcohol on AkEG21 binding to cellulose, AkEG21 (50 μg) was incubated with 10 mg of Bemcot in 1.0 mL of 50 mM sodium acetate buffer (pH 5.5) in the absence or presence of NaCl (0.5 or 1.0 M), ammonium sulfate (1.0 M), Triton X-100 (0.1 and 0.5%), SDS (0.1%) and ethanol (10%) at 25°C for 3 h. After washing five times with 1.0 mL of 50 mM sodium acetate buffer (pH 5.5) containing 0.1 M NaCl by centrifugation, the bound AkEG21 was eluted with 0.2 mL of the loading buffer at 95°C for 5 min and a 20 μL of aliquot was analyzed by SDS-PAGE.

The effect of carbohydrates on cellulose binding of AkEG21 was examined by incubating AkEG21 (50 μg) with Bemcot (5 mg) in 0.5 mL of 50 mM sodium acetate buffer (pH 5.5) in the absence or presence of 10 mg/mL glucose, cellobiose, maltose, soluble starch or laminaran, 1 mg/mL of cellohexaose or 5 mg/mL CMC at 25°C for 0.5 h. The reaction mixture was centrifuged, supernatant removed, and Bemcot was washed three times with 1.0 mL of 20 mM sodium acetate buffer (pH 6.0) containing 0.1 M NaCl. Bound AkEG21 was eluted with 0.1 mL of the loading buffer at 95°C for 5 min and 5 μL of sample was analyzed by SDS-PAGE.

To test the stability of AkEG21 binding to cellulose, AkEG21 (20 μg) was incubated with filter paper (10 mg) in 50 mM sodium acetate buffer (pH 5.5) at 4°C for 16 h. After washing with 50 mM sodium acetate buffer (pH 5.5) containing 0.1 M NaCl, filter paper was incubated in 0.1 mL of 50 mM sodium acetate buffer (pH 5.5) at 37°C for 24, 48, 72 and 96 h. AkEG21 binding was analyzed by SDS-PAGE.

### Quantitative analysis of AkEG21 cellulose binding activity

AkEG21 (0.1 mg) was incubated with increasing amounts (0–50 mg) of filter paper or Bemcot in 1.0 mL of 50 mM sodium acetate buffer (pH 5.5) at 25°C for 90 min. The reaction mixture was then centrifuged at 12,000 x g for 1 min, supernatant removed, and filter paper and Bemcot were washed five times with 1.0 mL of 20 mM sodium acetate buffer (pH 6.0) containing 0.1 M NaCl at 4°C for 10 min with continuous rotation followed by centrifugation. Finally filter paper and Bemcot were suspended in 0.2 mL of the loading buffer, heated at 95°C for 5 min and 20 μL aliquots were analyzed by SDS-PAGE. The intensity of the AkEG21 band was quantified using the Image J software version 1.50j (http://imagej.nih.gov/ij).

### Preparation of Anti-AkEG21 IgG

Rabbit antiserum against AkEG21 was generated by Eurofins Genomics Co (Tokyo, Japan) and anti-AkEG21 IgG was purified using AkEG21-immobilized Sepharose. The bound antibody was eluted with 20 mM glycine-HCl buffer (pH 2.5), neutralized with 1M Tris-HCl (pH 7.0) and dialyzed against phosphate-buffered saline (PBS).

### Immuostaining of AkEG21

AkEG21 binding to Bemcot was analyzed using the anti-AkEG21 antibody. Briefly, Bemcot fiber was mixed with 10 μg/mL AkEG21 in 50 mM sodium acetate buffer (pH 5.5) and incubated at 25°C for 2 h. As a control. Bemcot was incubated with the same concentration of BSA. Bemcot was washed three times with 1.0 mL of PBS by centrifugation and incubated with anti-AkEG21 IgG (0.14 μg/ml) in 1.0 mL PBS. After 16 h, Bemcot was washed four times with PBS and AkEG21 bound to cellulose fiber was visualized by incubation with Alexa Fluor 488-conjugated F(ab')_2_ fragment of goat anti-rabbit IgG H+L (0.2 μg/mL) for 16 h. After washing with PBS three times, fluorescence imaging was performed using an IN Cell Analyzer 6000 (GE Healthcare, Buckinghamshire, England).

Western blotting analysis of AkEG21 was performed using 5000-fold diluted anti-AkEG21 antiserum and 5000-fold diluted anti-rabbit IgG conjugated with horseradish peroxidase according to the manufacturer’s protocol. Immuno-reactive bands on the PVDF membrane were detected using 3,3’-diaminobenzidine tetrahydrochloride.

### Scanning electron microscopy (SEM)

Bemcot (10 mg) was incubated at 37°C for 24 h in the absence and presence of AkEG21 (0.1 mg) in 0.5 mL of 50 mM sodium acetate buffer (pH 5.5). Then, aliquots were analyzed by SEM. To characterize the hydrogel microstructures, the samples were freeze-dried and cut into small squares and coated with Pt/Pd using a sputter coater (E-1010, Hitachi, Tokyo, Japan). The samples were observed by Scanning electron microscopy (JCM6000-plus scanning electron microscope, JEOL Ltd., Tokyo, Japan) under an accelerating voltage of 1.5 kV.

### Synergistic effects of AkEG21 bound to cellulose with AkEG45 and 110K BGL on cellulose hydrolysis

Bemcot (10 mg) incubated with AkEG21 (50 μg) in 1.0 mL of 50 mM sodium acetate buffer (pH 5.5) at 4°C for 1 h was washed with four times with 1.0 mL of 20 mM sodium acetate buffer (pH 6.0) containing 0.1 M NaCl by centrifugation and designed as Bemcot-bound AkEG21. Bemcot-bound AkEG21 was incubated in the absence or presence of AkEG45 (10 μg) and /or 110K BGL (3.3 μg) in 1.0 mL of 50 mM sodium acetate buffer (pH 5.5) at 37°C. As a control, Bemcot (10 mg) was incubated with AkEG45 or/and 110K BGL in the absence and presence of AkEG21. After 24 h, the reaction products were analyzed by TLC [28] as mentioned above. The synergistic effect of AkEG21 with AkEG45 on Bemcot digestion was further examined as follows. Bemcot (10 mg) was incubated with 10, 25, 50, 75, 100, 150, 200 μg of AkEG21in the absence and presence of AkEG45 (10 μg) in 1.0 mL of 50 mM sodium acetate buffer (pH 5.5) at 37°C for 20 h. Reducing sugar and total hexose liberated from the substrate were determined by the method of Nelson and Somogyi [27] and the phenol-sulfuric acid method [29], respectively. Filter paper (10 mg) was incubated with AkEG21 (0, 0.1, 0.2, 0.3 mg) in the absence or presence of AkEG45 (4.6, 9.4, 18.6, 37.2, 55.8 μg) in 1.0 mL of 50 mM sodium acetate buffer (pH 5.5) for 20 h and the reducing sugar liberated from the filter paper was determined. CMC (10 mg) was digested with increasing amounts of AkEG21 (0–80 μg) in the absence and presence of AkEG45 (0.75 μg) at 37°C; the reaction was terminated after 20 min by incubation at 95°C for 5 min. The amount of reducing sugar liberated from CMC was determined.

### Synergistic effects of AkEG21 and CBHs on cellulose hydrolysis

Filter paper (10 mg) was incubated with CBH 1 or CBH 2 (10 μg) in the absence or presence of AkEG21 (25 μg) in 1.0 mL of 50 mM acetate buffer (pH 5.5) containing 0.1 M NaCl at 37°C for 24 h. As a control, filter paper was incubated with AkEG21 without CBHs. To confirm the synergistic action of AkEG21 and CBH 2, the filter paper was incubated with increasing amounts of AkEG21 (0–80 μg) in the absence or presence of CBH 2 (10 μg) at 37°C for 20 h. The amount of reducing sugar liberated from filter paper was determined. The experiments were repeated four times.

### Removal of AkEG21 from the digestive fluid of sea hare by immunoprecipitation

AkEG21 was removed by immunoprecipitation with an AkEG21-specific polyclonal antibody as follows: Frozen digestive fluid (55 mL) of sea hare was thawed and fractionated with ammonium sulfate at 30%-60% saturation. The resulting precipitate was dissolved in 20 mM sodium phosphate buffer (pH 7.0) containing 0.15M NaCl and dialyzed against the same buffer. The dialysate was centrifuged at 12,000 x g for 10 min, and the supernatant (protein concentration: 6.9 mg/mL, 13 mL) was used as a concentrated digestive fluid of sea hare. The concentrated digestive fluid (0.5 mL) was incubated with purified anti-AkEG21 IgG (1.0 mg) and incubated at room temperature for 16 h. As a control, the digestive fluid was incubated with the same volume of PBS. After centrifugation at 12,000 x g for 10 min, the supernatant was incubated with 60 μL of protein G-Sepharose gel and incubated at 4°C for 30 min with occasional mixing to remove the soluble AkEG21-Ab complex and non-reacted anti-AkEG21 IgG. The immunoprecipitate obtained by centrifugation was washed three times with 1.0 mL of TNE buffer (20 mM Tris-HCl buffer [pH 7.0] containing 0.15M NaCl, 0.1% NP-40 and 1 mM EDTA) by centrifugation. The washed immunoprecipitate was analyzed by SDS-PAGE to examine the amount of AkEG21 in the immunoprecipitate. Immunoprecipitation of the supernatant and protein G-Sepharose treatment was repeated two more times. Cellulase activity toward CMC and Bemcot of the supernatants obtained by first and third immunoprecipitation were assayed.

## Results

### Screening of cellulose binding proteins in the digestive fluid of sea hare

**Fig 1:**
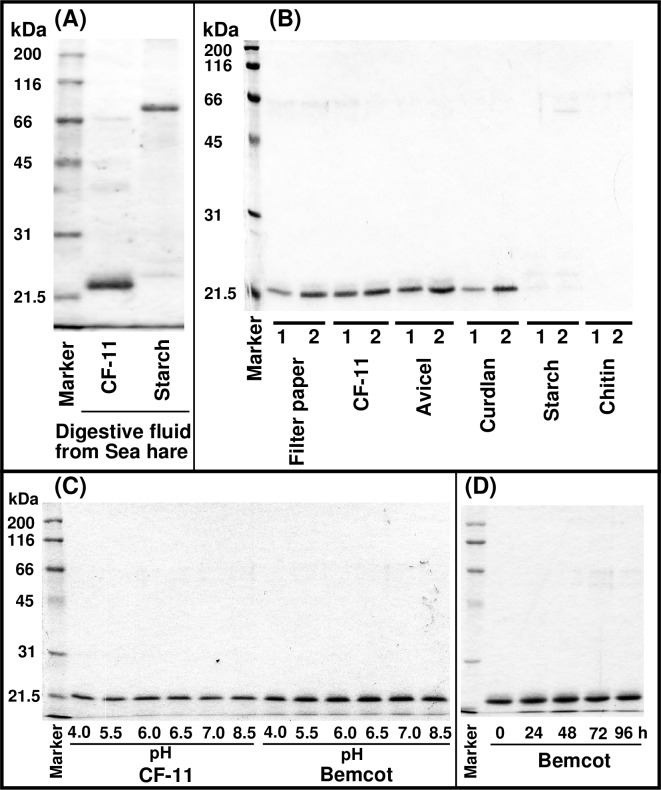
Cellulose-binding activity of AkEG21. **(**A) Screening of cellulose binding proteins in the digestive fluid of sea hare. The digestive fluid was incubated with CF-11 or starch and the bound proteins were analyzed by SDS-PAGE. (B) Binding specificity of AkEG21. AkEG21 was incubated with filter paper, CF-11, Avicel, curdlan, starch and chitin, eluted with the loading buffer in the presence (1) or absence (2) of ß-mercaptoethanol and analyzed by SDS-PAGE. (C) Effect of pH on AkEG21 cellulose-binding activity. AkEG21 was incubated with CF-11 or Bemcot at the indicated pH and the binding was analyzed by SDS-PAGE. (D) Stability of AkEG21 binding to cellulose. Bemcot bound to AkEG21 was incubated in 50 mM sodium acetate buffer (pH 5.5) at 37°C for the indicated time and the bound AkEG21 was determined by SDS-PAGE.

Here, we attempted to isolate proteins enhancing enzymatic cellulose digestion by screening for cellulose binding activity in the *A*. *kurodai* digestive fluid which was incubated with cellulose fiber (CF-11) or semi-crystalline starch used as control. SDS-PAGE analysis revealed 21-kDa and 80-kDa proteins bound to cellulose and starch, respectively (Fig 1A), which were identified as ß-1,4 endoglucanase (AkEG21) and α-amylase (ApAmy80) based on exact similarity of N-terminal amino acid sequences EQKCQPNSHGVRMYQ [22] and ASAYHDP [33], respectively. Although AkEG21 does not possess a CBD [23], the enzyme showed strong binding to CF-11, filter paper, Avicel and ß-1,3-glucan curdlan, but not to starch and chitin (Fig 1B), indicating that AkEG21 has specific affinity to ß-1, 4 and ß-1,3 glucans.

Next we examined the effect of pH on the binding of AkEG21 to cellulose using CF-11 and Bemcot. AkEG21 was bound to CF-11 and Bemcot in the broad pH range (Fig 1C) and its affinity to Bemcot was higher than that of CF-11. As shown in Fig 1D, the dissociation of AkEG21 from Bemcot was not detected even after incubation at 37°C for 96 h, indicating that AkEG21 binding to cellulose is highly stable.

### Screening of cellulose binding proteins among proteins secreted by *T*. *viride*

**Fig 2:**
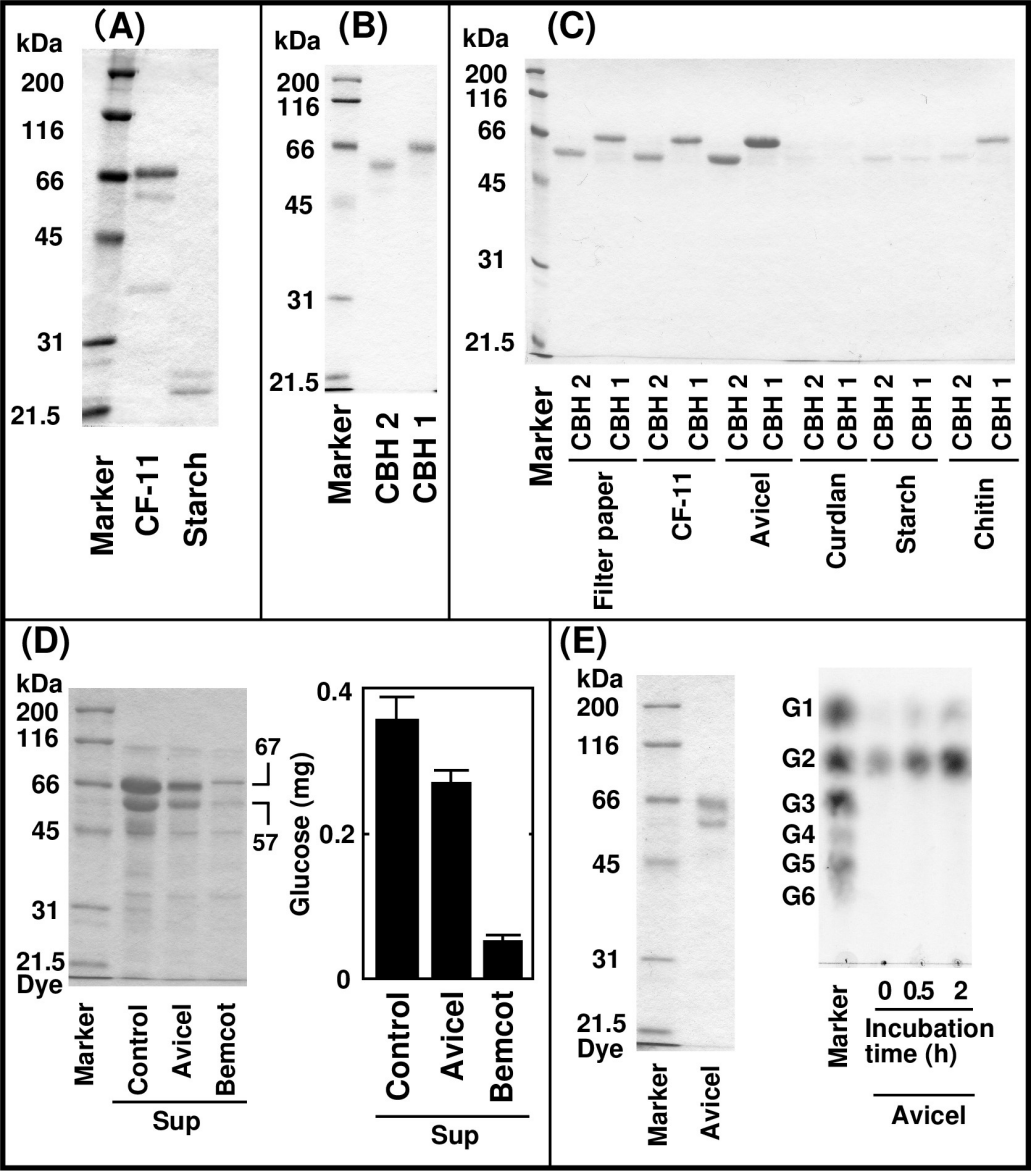
Cellulose-binding proteins in Meicelase. (A) Screening of cellulose-binding proteins in Meicelase. Identification of cellulose-binding proteins was performed by incubating Meicelase with CF-11 or starch and the bound proteins were analyzed by SDS-PAGE. (B) CBH 1 and 2 were purified from Meicelase and analyzed by SDS-PAGE. (C) Binding specificity of CBH 1 and 2 was examined by incubation with filter paper, CF-11, Avicel, curdlan, starch and chitin followed by SDS-PAGE. (D) Removal of 67- and 57-kDa proteins from Meicelase by reaction with Avicel or Bemcot (left) and its effect on glucose producing activity from filter paper of Meicelase (right). Incubation of Meicelase with Avicel or Bemcot and assay of glucose-producing activity from filter paper by the supernatants were carried out as described in Materials and Methods. Average values for the triplicate measurements are shown with standard deviation (right). (E) Detection of cellobiohydrolase activity of the 67- and 57-kDa proteins bound to Avicel. Cellobiohydrolase activity was detected as described in Materials and Methods.

The cellulolytic system of *T*. *viride* is similar to that of *T*. *reesei* [34, 35], which consists of three types of glucanases, endoglucanases, CBHs, ß-glucosidases and swollenin [3, 4, 19, 34, 35]. Secreted proteins from *T*. *viride* were used as an industrial cellulase cocktail (Meicelase). Many details about the structure and function of these cellulases have been well elucidated [1–3] and many glycosidases possessing a cellulose-binding domain such as CBHs and non-catalytic cellulose-binding proteins have been identified. In order to compare cellulose-binding proteins in *A*. *kurodai* digestive fluid and *T*. *viride* secreted proteins, the same method applied to the screening of the *A*. *kurodai* cellulose-binding proteins was used for Meicelase. As a result, three cellulose binding proteins (67-, 57- and 37-kDa), which did not bind starch, were identified; among them, the 67-kDa protein was the most prominent (Fig 2A). However, N-terminal sequencing performed for further identification of the cellulose-binding proteins was unsuccessful, suggesting that N-termini were blocked. As previous reports indicated that CBH 1 and 2 had molecular masses of about 67 and 57 kDa, respectively and that their N-termini were also blocked [3, 4], we purified CBH 1 and 2 from Meicelase (Fig 2B) and confirmed their ability to bind cellulose (Fig 2C). Similar to AkEG21, CBH 1 and 2 demonstrated binding to filter paper, CF-11 and Avicel; however, in contrast to AkEG21, CBH 1 showed weak affinity to chitin. These results suggest that 67 and 57 kDa proteins are CBH 1 and 2, respectively. The identity of the 37-kDa cellulose-binding protein remains uncertain.

In order to confirm the identities for the 67 and 57 kDa proteins, glucose producing activity from filter paper of Meicelase treated with Avicel or Bemcot and reaction products liberated from Avicel or Bemcot bound to 67- and 57-kDa proteins were analyzed. When Meicelase was incubated with Avicel or Bemcot, 67- and 57-kDa proteins in the supernatant were greatly decreased (Fig 2D, left). Densitometric analysis indicated that 67 kDa protein band was decreased to 53% and 17% of control by Avicel and Bemcot treatment, respectively. The 57 kDa protein band was decreased 54% and 17%. ß-Glucosidase activity of control Meicelase, Avicel- or Bemcot-treated Meicelase were 19.7 ± 0.6, 18.9 ± 2.8 and 16.0 ± 3.0 μmol/min/mL, respectively. Thus, Avicel or Bemcot treatment had little or weak effect on ß-glucosidase activity, whereas glucose producing activity from filter paper was more strongly affected by treatment with Avicel or Bemcot than ß-glucosidase activity (Fig 2D, right). Especially, the activity was markedly decreased (15% of control) by Bemcot treatment. Next, the enzyme activity of Avicel bound to 67- and 57-kDa proteins were analyzed. Like the case of CF-11, 67- and 57-kDa proteins were major binding proteins to Avicel (Fig 2E, left). When Avicel bound to 67- and 57-kDa proteins was incubated without external substrate, cellobiose was produced as a major product by time dependent manner (Fig 2E, right). Trace amount of glucose monomer was also produced, suggesting that cellobiose was cleaved to glucose monomer by ß-glucosidase slightly bound to Avicel.

Taken together these results, it is highly likely that 67- and 57-kDa proteins bound to cellulose are identical to CBH 1 and 2.

### Comparison of sequence of AkEG21, GH45 endoglucanases and cellobiohydrolases

**Fig 3:**
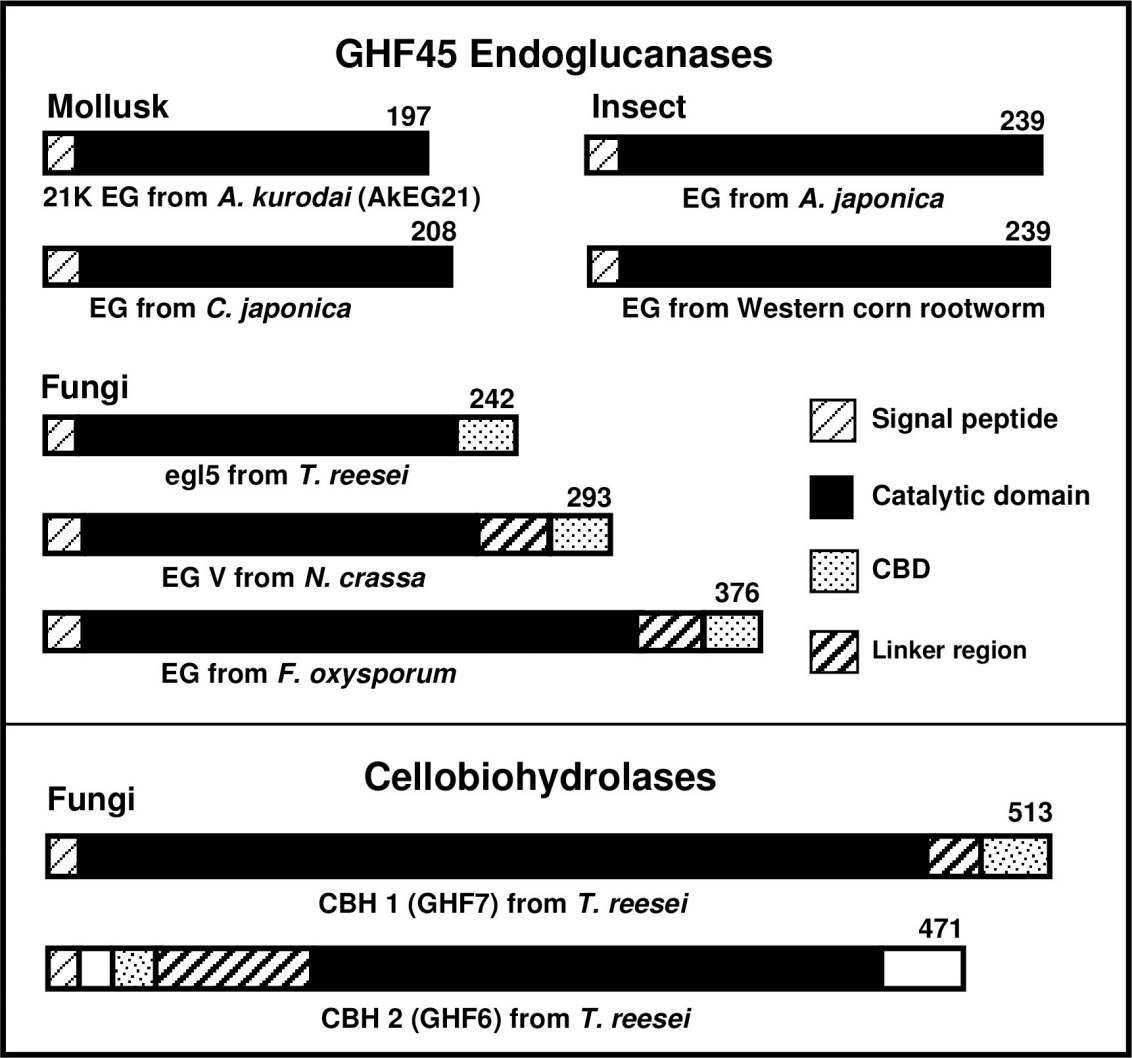
Domain structures of GH45 endoglucanases (EGs) from mollusks, insects and fungi and cellobiohydrolases (CBHs) from fungi. AkEG21 (21-kDa EG from *A*. *kurodai* (UniProt accession number A0A077JGH0), EGs from *C*. *japonica* (B9X0W0), *A*. *japonica* (X2D6C4) and Western corn rootworm (I1VZE8), egl5 from *T*. *reesei* (P43317), EG V from *N*. *crassa* (Q1K5M0), EG from *F*. *oxysporum* (P45699), and CBH 1 (P62694) and CBH 2 (P07987) from *T*. *reesei*. CBD: cellulose-binding domain.

The 197-amino acid sequence of AkEG21 comprises an N-terminal signal peptide and a 180 residue catalytic domain [23] which is highly homologous to those of GHF45 cellulases identified in other mollusks such as blue mussel (*Mytilus edulis*) [36], giant snail (*Ampullaria crossean*) [37], disc abalon (*Haliotis discus*, UniProt: accession number B6RB06) and brackish water clam (*Corbicula japonica*) [38]. We compared domain structures of GHF45 cellulases from mollusks, insects, fungi, and CBH 1 and 2 from *T*. *reesei* (Fig 3). GHF 45 cellulases from mollusks (AkEG21 [23] and EG from *Corbicula japonica* [38]) and insects (EGs from *Apriona japonica* [24] and Western corn rootworm [39]) do not possess CBDs, whereas those from fungi (egl5 from *T*. *reesei* [40], EGV from *Neurospora crassa* [41], EG from *Fusarium oxysporum* [42]) have CBDs of various sizes. Although the length of GHF45 cellulases from mollusks and insects and *T*. *reesei* (egl5, 242 residues) are similar, the latter contains three conserved regions: the catalytic domain, CBD, and linker connecting the CBD with catalytic domain [40]. CBH 1 and 2 belong to GHF7 and GHF6, respectively [43, 44]. The CBD of CBH 1 is located at the C-terminus, whereas that of CBH 2 is at the N-terminus [3]. No regions homologous with the CBD of fungal GHF45 cellulases and CBHs were identified in mollusk GHF 45 cellulases, suggesting that in AkEG21, the cellulose-binding site is located within its catalytic domain.

Recently lytic polysaccharide monooxygenase (LPMO) was shown to be an important enzyme for the decomposition of lignocellulose and chitin in fungi and bacteria [45]. Most LPMOs belonging to GHF61 are composed of approximately 200 amino acid residues like mollusk GH45 endoglucanase. No sequence homology between AkEG21 and LPMOs was found.

A cellulose binding protein expansin has N-terminal and C-terminal domains [46] homologous to the catalytic domain of GHF45 cellulases and the CBD of cellulases, respectively. As in AkEG21 no sequence homology to the expansin CBD was found, we aimed to clarify why AkEG21 lacking the CBD exhibited strong cellulose-binding activity similar to that of CBHs.

### Characterization of AkEG21 cellulose-binding activity

**Fig 4:**
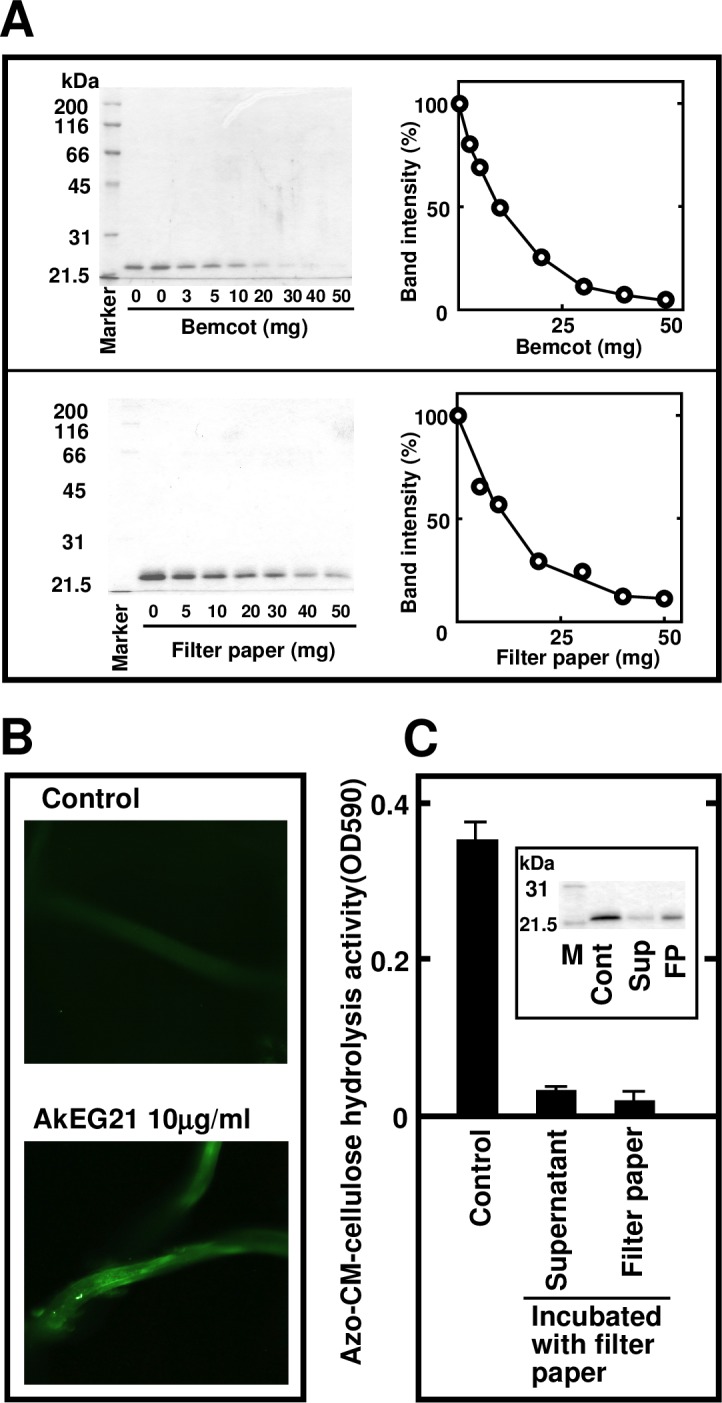
Analysis of AkEG21 affinity to cellulose. (A) Quantification of AkEG21 binding to cellulose. AkEG21 was incubated with increasing amounts of Bemcot or filter paper and cellulose-bound AkEG21 was analyzed by SDS-PAGE and quantified by ImageJ. The experiment was repeated twice with similar results. (B) Fluorescence image of AkEG21 bound to cellulose. Washed Bemcot cellulose fibers were incubated with BSA (control) or AkEG21 and immunostained with the anti-AkEG21 IgG as described in Materials and Methods. (C) Cellulase activity of filter paper-bound AkEG21 toward an external substrate azo-CMC. The activity is shown as mean ± standard deviation from three separate determinations. The amounts of AkEG21 in the reaction was analyzed by SDS-PAGE (inset).

We first examined the kinetics of AkEG21 cellulose-binding activity toward Bemcot and filter paper by incubating AkEG21 (0.1 mg) with different amounts of Bemcot or filter paper. Almost all AkEG21 was bound to 30 mg of Bemcot and 50 mg of filter paper (Fig 4A), indicating that Bemcot is a better substrate for AkEG21 binding than filter paper.

The binding of AkEG21 to Bemcot was visualized by immunofluorescence using anti-AkEG21 antibody (Fig 4B), further confirming the specificity of AkEG21 affinity to cellulose fibers. The enzymatic activity of filter paper-bound AkEG21 toward an external substrate was determined using azo-CMC. Although most AkEG21 was bound to filter paper, its activity toward azo-CMC was very low (Fig 4C), suggesting that cellulose-bound AkEG21 does not hydrolyze external substrates.

**Fig 5:**
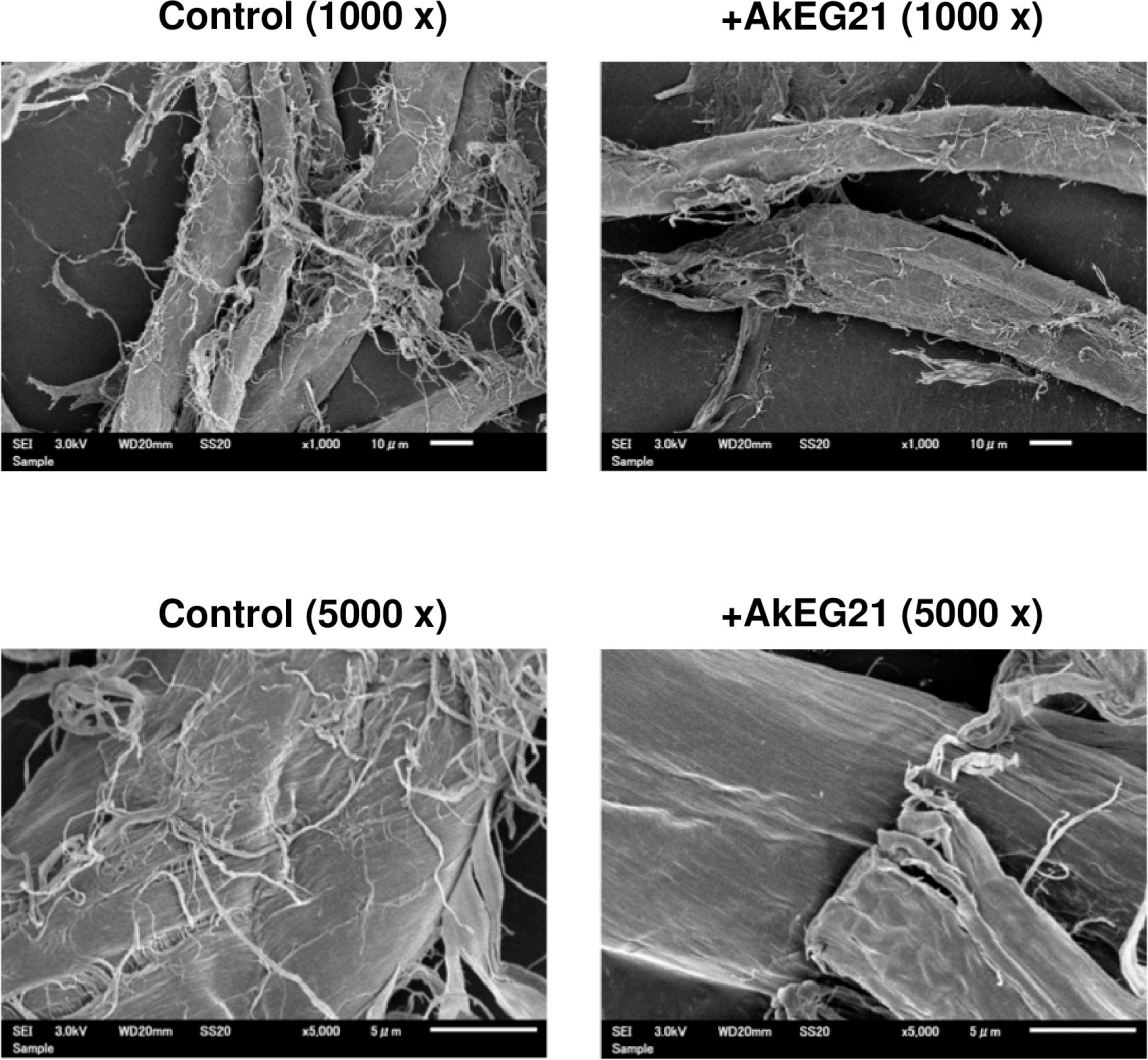
Surface structure of cellulose fibers treated with AkEG21. SEM images of cellulose fibers (control) and AkEG21-treated cellulose fibers at a magnification of 1000x and 5000x. Bemcot was used as cellulose fibers.

SEM was carried out to observe the morphological changes of cellulose fibers by incubation with AkEG21 (Fig 5). It was obvious that the treated samples have more slender fibers and the stem was surrounded by many free filamentous fibers. However, the major structural difference between control and AkEG21-treated cellulose fibers was not observed.

**Fig 6:**
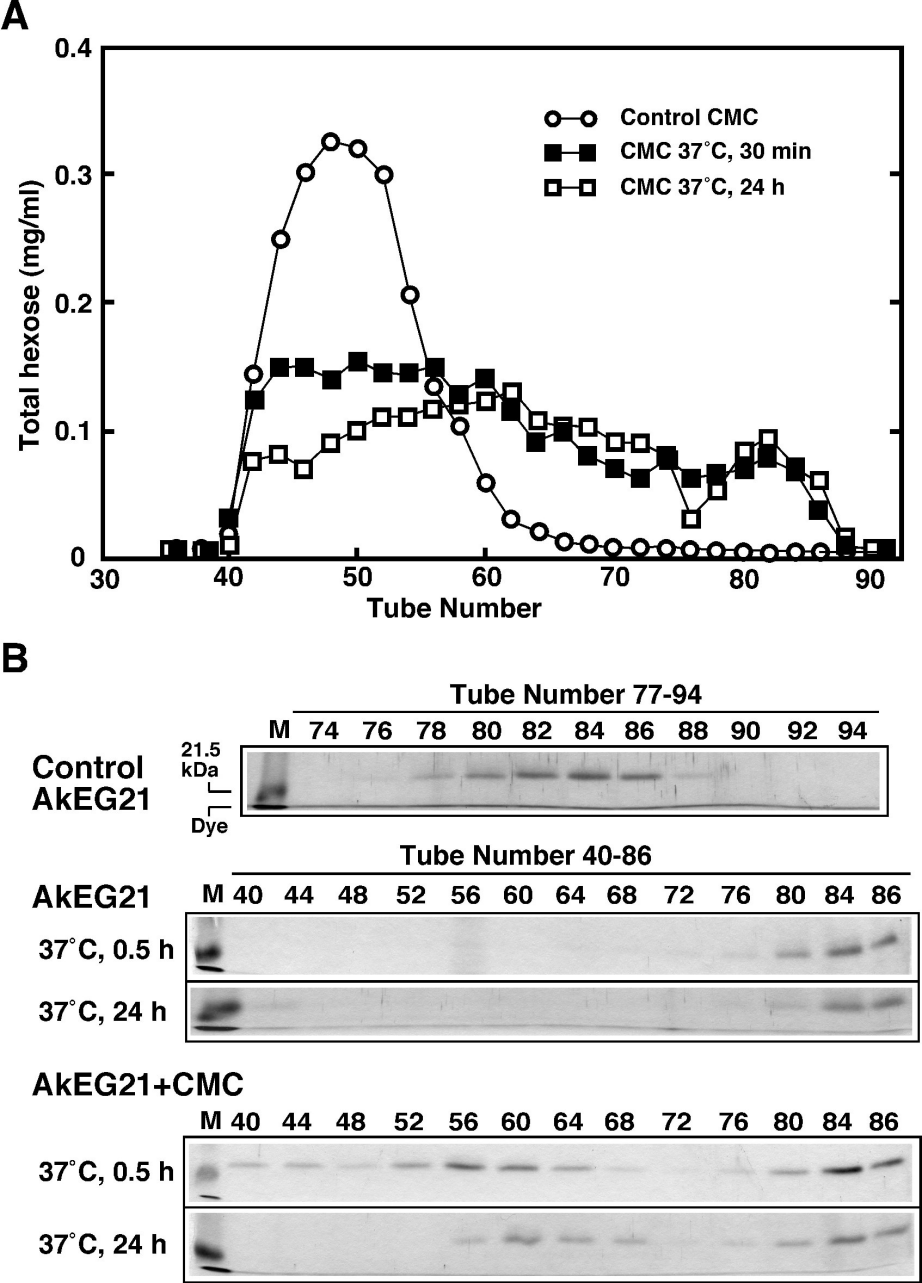
Interaction of AkEG21 with carboxymethylcellulose (CMC). (A) CMC was incubated with AkEG21, reaction products were separated by gel filtration and total hexose in the eluate was determined as described in Materials and Methods. (B) The eluate (0.1 mL) was mixed with 20 μL of 6 x loading buffer and treated at 95°C for 5 min. The sample (20 μL) was separated by 12% SDS-PAGE and AkEG21 was detected by silver staining. Elution profiles of AkEG21 incubated at 37°C for 30 min or 24 h in the absence of CMC were also analyzed as controls.

Next, we examined AkEG21 binding to CMC using gel filtration (Fig 6). AkEG21 incubated with CMC at 37°C for 30 min or 24 h and AkEG21 alone were applied to a Sephacryl S-100 column; thereafter, the elution profiles of the CMC reaction products and AkEG21 were analyzed. The digestion profiles of CMC by AkEG21 were analyzed by measuring the total hexose in the eluate. Control CMC was mostly eluted in tubes numbered 40–60, whereas CMC was digested by AkEG21 to small-molecular weight products of various length (Fig 6A). Although AkEG21 alone was mainly eluted in fraction numbers 80–86, AkEG21 incubated with CMC was eluted in the higher molecular weight fraction in addition to the same fraction of AkEG21 alone (Fig 6B). The aggregation of AkEG21 by incubation at 37°C for 30 min or 24 h did not occur, whereas the high molecular weight AkEG21 was detected in fractions 40–68 and fractions 56–68 by incubation with CMC for 30 min and 24 h, respectively. By increasing the reaction time with CMC, the elution position of high molecular weight AkEG21 was shifted to a lower molecular weight. These results indicate that AkEG21 could bind to CMC as well as to cellulose and AkEG21 remained in a complex with the digested fragment of CMC even after incubation at 37°C for 24 h.

**Fig 7:**
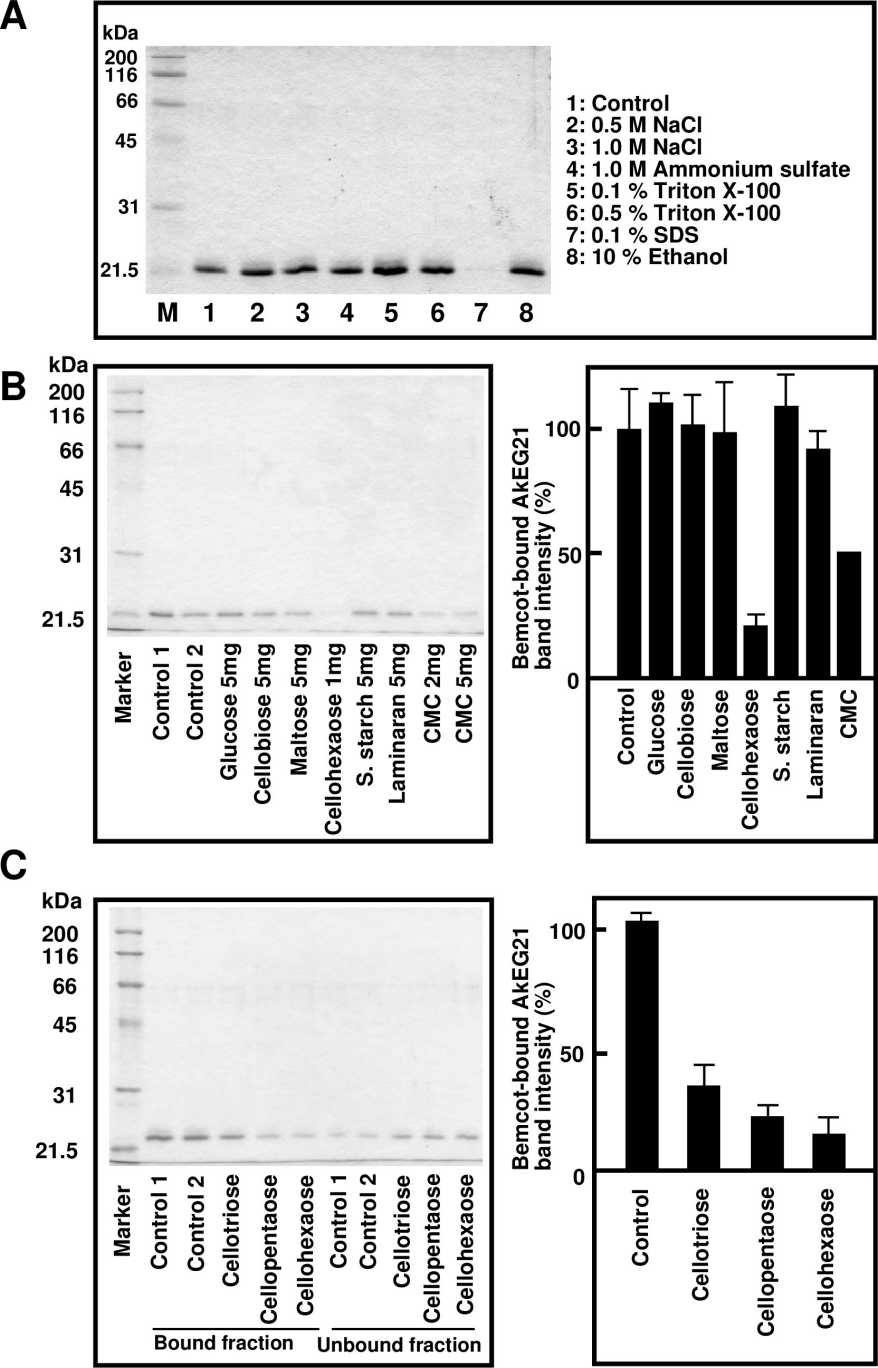
Characterization of AkEG21 cellulose-binding activity. (A) Effect of ionic strength, detergents and alcohol on AkEG21 binding to cellulose. AkEG21 was incubated with Bemcot in the absence or presence of NaCl (0.5 or 1.0 M), ammonium sulfate (1.0 M), Triton X-100 (0.1 and 0.5%), SDS (0.1%), and ethanol (10%), and AkEG21 binding to Bemcot was analyzed by SDS-PAGE. Repeat experiments yielded similar results. (B) Effect of carbohydrates on cellulose binding of AkEG21. AkEG21 was incubated with Bemcot in the absence or presence of glucose, cellobiose, maltose, cellohexaose, soluble starch (S. starch), laminaran, or CMC. The relative band intensity (mean ± standard deviations) was calculated from four separate experiments using ImageJ. (C) Effect of oligocellulose chain length on AkEG21 binding to cellulose. AkEG21 was incubated with Bemcot in the absence or presence of cellotriose, cellopentaose or cellohexaose (1 mg/mL) and AkEG21 binding to Bemcot was analyzed by SDS-PAGE. The relative band intensity (mean ± standard deviation) was calculated from three separate experiments using ImageJ.

To determine the binding mode of AkEG21 to cellulose, we used different inhibitory conditions. As shown in Fig 7A, AkEG21 affinity to cellulose was not affected by the treatment with high concentration of NaCl, ammonium sulfate, nonionic detergent (0.5% Triton X-100) and 10% ethanol, but was completely inhibited by SDS. We then examined the effect of glucose, maltose, cellobiose, cellotetraose and polysaccharides on AkEG21 interaction with cellulose. The intensity of Bemcot-bound AkEG21 bands on SDS-PAGE were compared. The results indicated that the binding of AkEG21 to cellulose was not altered by glucose, cellobiose, maltose, starch (soluble) and laminaran, but was significantly reduced by cellohexaose and to some extent by CMC (Fig 7B).

The effect of oligocelluloses with different chain length on AkEG21 binding to cellulose fibers was assessed by incubating AkEG21 with cellotriose, cellopentaose and cellohexaose. Among them, cellohexaose showed the strongest and cellotriose the weakest inhibition of AkEG21-cellulose interaction (Fig 7C) which is consistent with our previous report that cellohexaose was a good substrate for AkEG21, whereas cellotriose was not cleaved [22]. These results strongly suggested that cellohexaose and cellulose compete cellulose binding site of AkEG21. Cellotriose might bind to cellulose binding site of AkEG21 more weakly than cellohexaose although it is not cleaved by AkEG21.

### Enzymatic activity of cellulose-bound AkEG21

**Fig 8:**
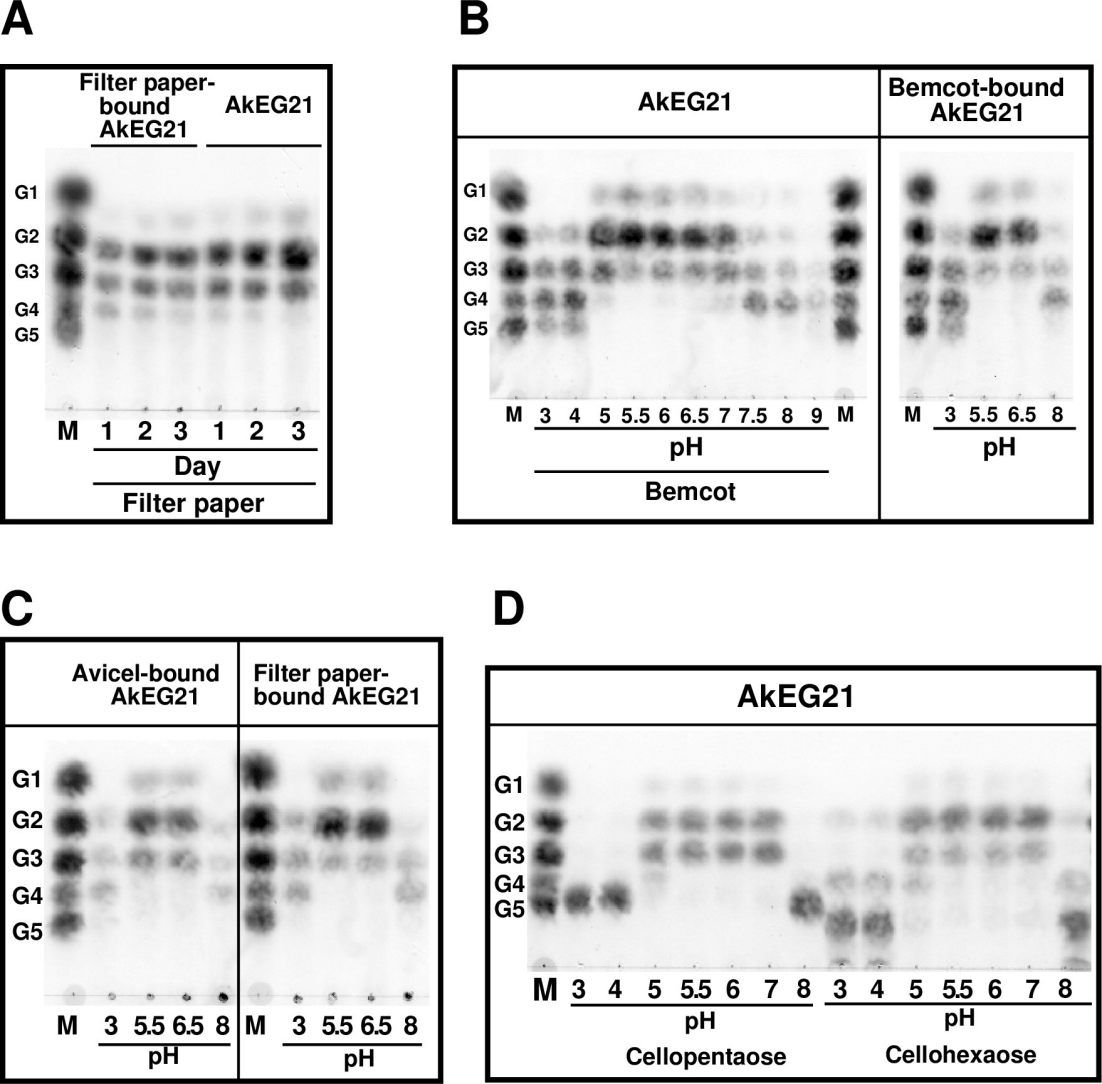
Characterization of cellulase activity of cellulose-bound AkEG21. Filter paper- and Bemcot-bound AkEG21 were prepared as described in Materials and Methods and incubated without addition of AkEG21. (A) Filter paper-bound AkEG21 was incubated in the reaction buffer for the indicated time. As a control, filter paper was incubated with AkEG21 (50 μg) without separation of free and filter paper-bound AkEG21. (B-D) Effect of pH on cellulose activity. Sodium acetate buffer (50 mM, pH 3.0–6.5) and Tris-HCl buffer (50 mM, pH 7.0–9.0) were used. (B) Effect of pH on cellulase activity of Bemcot-bound AkEG21. AkEG21 bound to Bemcot was incubated at a broad pH (right). As a control, Bemcot was incubated with AkEG21 (50 μg) without separation of free and Bemcot-bound AkEG21 (left). (C) Effect of pH on cellulase activity of Avicel- and filter paper-bound AkEG21. (D) Effect of pH on AkEG21 activity toward oligocelluloses. Cellopentaose and cellohexaose (20 μg) were digested with AkEG21 (1 μg) at the indicated pH for 1 h at 37°C. In all experiments, the reaction products were analyzed by TLC. TLC was repeated three times and similar results were obtained.

As AkEG21 showed tight binding to cellulose fibers without dissociation (Fig 1D), we analyzed the catalytic activity of cellulose-bound AkEG21. Filter paper-bound AkEG21 produced cellobiose and cellotriose as major and cellotetraose and glucose as minor hydrolysis products, which was similar to the effect of free AkEG21 incubated with filter paper (Fig 8A). These results indicate that cellulose-bound AkEG21 could hydrolyze cellulose as an internal substrate like CBH [7, 8].

If AkEG21 binds to cellulose via the catalytic domain, the affinity of AkEG21 should be affected by pH. Our results showed that AkEG21 exhibited cellulose binding at a broad pH range (4–8.5) (Fig 1C) and a previous report indicated that the optimal pH for AkEG21 activity toward CMC was about 4.3–5.6 [23]. AkEG21 retained approximately 50% and 10% of the maximum activity at pH 3 and pH 8, respectively; however, the effect of pH on cellulose hydrolysis by cellulose-bound AkEG21 was not examined. To determine it, AkEG21 bound to Bemcot was incubated at various pH and the reaction products were analyzed by TLC (Fig 8B, right); as a control, Bemcot was incubated with AkEG21 without separation of free and cellulose-bound AkEG21 (Fig 8B, left). The profiles of cello-oligosaccharides produced from Bemcot were almost the same in both reaction systems; furthermore, at pH 5.0–6.5, cellobiose was a major hydrolysis product of Bemcot and filter paper. The pH profiles of hydrolysis products of Avicel, filter paper and Bemcot were very similar (Fig 8B and 8C). However, cellopentaose, cellotetraose and cellotriose were mostly produced at pH 3.0–4.0, whereas cellotetraose and cellotriose were produced at pH >7.5.

Next, we examined the effect of pH on AkEG21 activity toward cellopentaose and cellohexaose (Fig 8D). Cellopentaose was digested by AkEG21 to cellotriose and cellobiose at equal propotions at pH 5–7, but no hydrolysis occured at pH 3–4 and pH 8. Cellohexaose was hydrolyzed mostly to cellobiose followed by cellotriose at pH 5–7, and to cellotetraose and cellobiose at pH 3–5 and pH 8, although in the latter reactions the relative amount of digestion products was low.

Thus, compared to effects of pH on cello-oligosaccharide hydrolysis by AkEG21, pH dependence of the activity of AkEG21 toward cellulose is broader: AkEG21 bound to cellulose remains active at pH 3.0 and pH 8.0. Overall, these findings indicate that AkEG21 binding to cellulose is active at a broad pH range, which is consistent with AkEG21 binding to cellulose via the substrate-binding site within the catalytic domain.

### Synergistic effects of AkEG21 with AkEG45 and 110K BGL on cellulose hydrolysis

**Fig 9:**
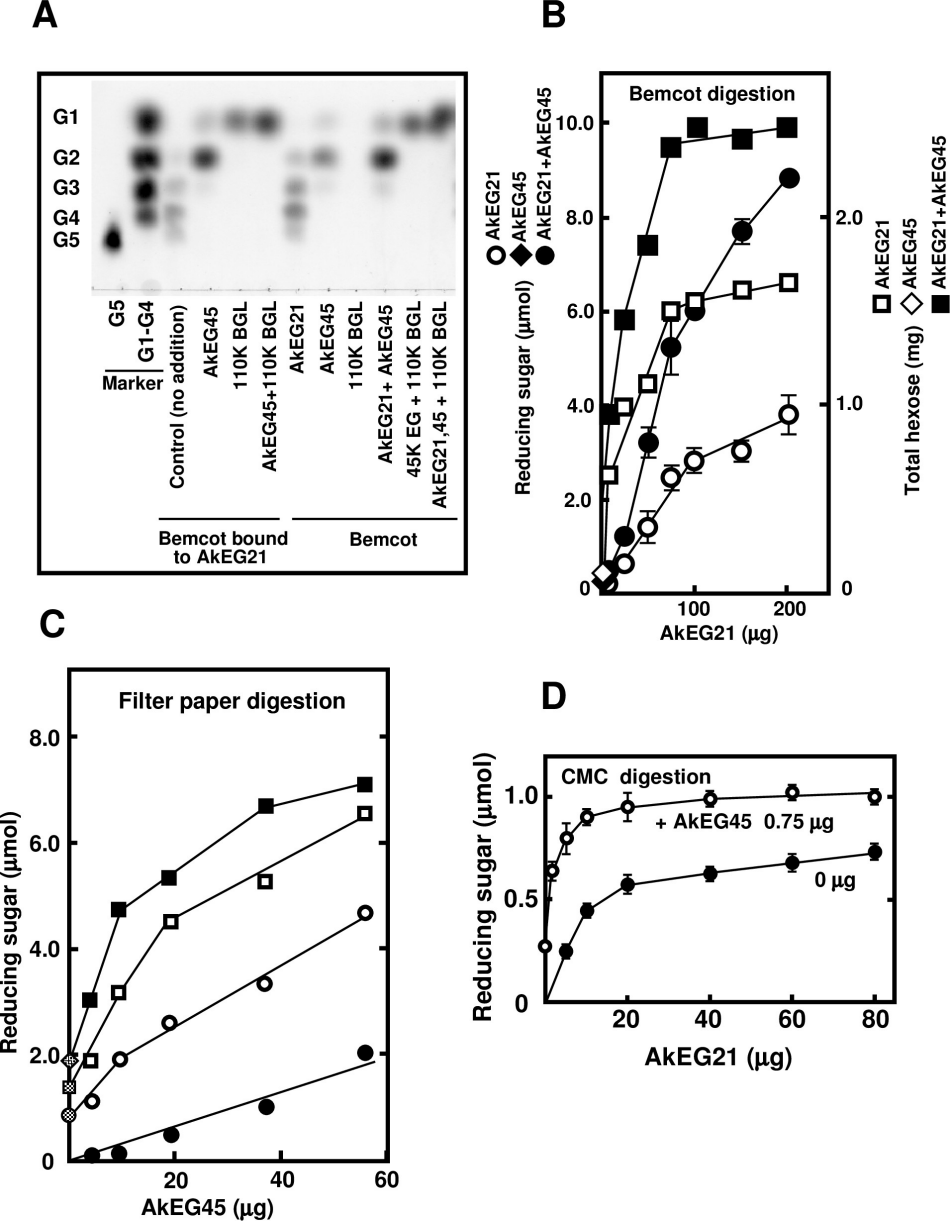
Hydrolysis of Bemcot and filter paper by the synergistic activity of AkEG21 and other glycosidases. (A) Synergy between cellulose-bound AkEG21, AkEG45 and 110K BGL. Bemcot-bound AkEG21 was incubated in the absence or presence of AkEG45 and /or 110K BGL at 37°C for 24 h and reaction products was analyzed by TLC. (B-D) Kinetics of AkEG21 and AkEG45 synergy. Cellulose hydrolysis was assessed based on the release of total hexose and reducing sugar. (B) Bemcot was digested with increasing amounts of AkEG21 in the absence (open circles and squares) or presence (closed circles and squares) of AkEG45 (10 μg) at 37°C for 20 h. After reactions, reducing sugar (circles) and total hexose (squares) liberated from Bemcot were determined. The activities of AkEG45 are shown as closed (reducing sugar) and opened (total hexose) diamond on the Y-axis. Average values of the reducing sugar liberated from three separate experiments are shown with standard deviation. Average values of the total amount of hexose liberated for the duplicate measurements are shown and similar results were obtained. (C) Filter paper was digested with increasing amounts of AkEG45 in the absence (closed circles) or presence (0.1 mg: opened circles, 0.2 mg: opened squares, 0.3 mg: closed squares) of AkEG21 at 37°C for 20 h and reducing sugar liberated from filter paper was determined. The amount of reducing sugar released by AkEG21 only are shown as hatched circle (0.1 mg AkEG21), hatched square (0.2 mg) and hatched diamond (0.3 mg). The experiment was repeated twice and similar results were obtained. Average values of reducing sugar liberated are shown. (D) CMC (1%) was digested with increasing amounts of AkEG21 in the absence (closed circles) or presence (open circles) of AkEG45 (0.75 μg) at 37°C for 20 min and reducing sugar liberated from CMC was determined. The data (mean ± standard deviation) were calculated from three separate experiments.

To analyze synergy between cellulose-bound AkEG21 and other cellulases of *A*. *kurodai* on cellulose hydrolysis, Bemcot-bound AkEG21 was incubated with AkEG45 and / or 110K BGL; Bemcot without AkEG21 was used as control (Fig 9A). When Bemcot-bound AkEG21 was incubated with AkEG45, the production of cellobiose was markedly increased compared to the control. No reaction products were detected when Bemcot alone was incubated with 110K BGL, but glucose was detected in high amounts in the reaction containing Bemcot-bound AkEG21 and 110K BGL and especially in that also containing AkEG45. These results indicate that cellobiose released from cellulose by AkEG21 and AkEG45 can be further converted to glucose by the activity of 110K BGL.

The synergistic effects of AkEG21 and AkEG45 toward hydrolysis of cellulose and CMC were evaluated by the production of reducing sugar and total hexose. For this, Bemcot was incubated with increasing concentrations of AkEG21 in the absence or presence of AkEG45 (Fig 9B). Although the activity of AkEG45 (10 μg) alone is very low (reducing sugar: 0.206 ± 0.19 μmol, total hexose: 0.337± 0.01 μmol), the release of both reducing sugar and hexose due to AkEG21 hydrolysis of Bemcot was markedly increased by the addition of AkEG45; these levels were higher than those produced by the sum of the individual activities of AkEG21 and AkEG45. For example, reducing sugar producing activity was increased by approximately two-fold compared to the sum of the individual activities of AkEG21 and AkEG45 when Bemcot was hydrolyzed by combination of AkEG21 (100 μg) and AkEG45 (10 μg). A similar synergistic effect was observed also for hydrolysis of filter paper (Fig 9C). In contrast, no synergy between AkEG21 and AkEG45 was detected in CMC hydrolysis (Fig 9D). CMC hydrolysis by combination of AkEG21 and AkEG45 is almost equal to the sum of the activity of AkEG21 and AkEG45.

### Synergistic effects of AkEG21 and CBHs on cellulose hydrolysis

**Fig 10:**
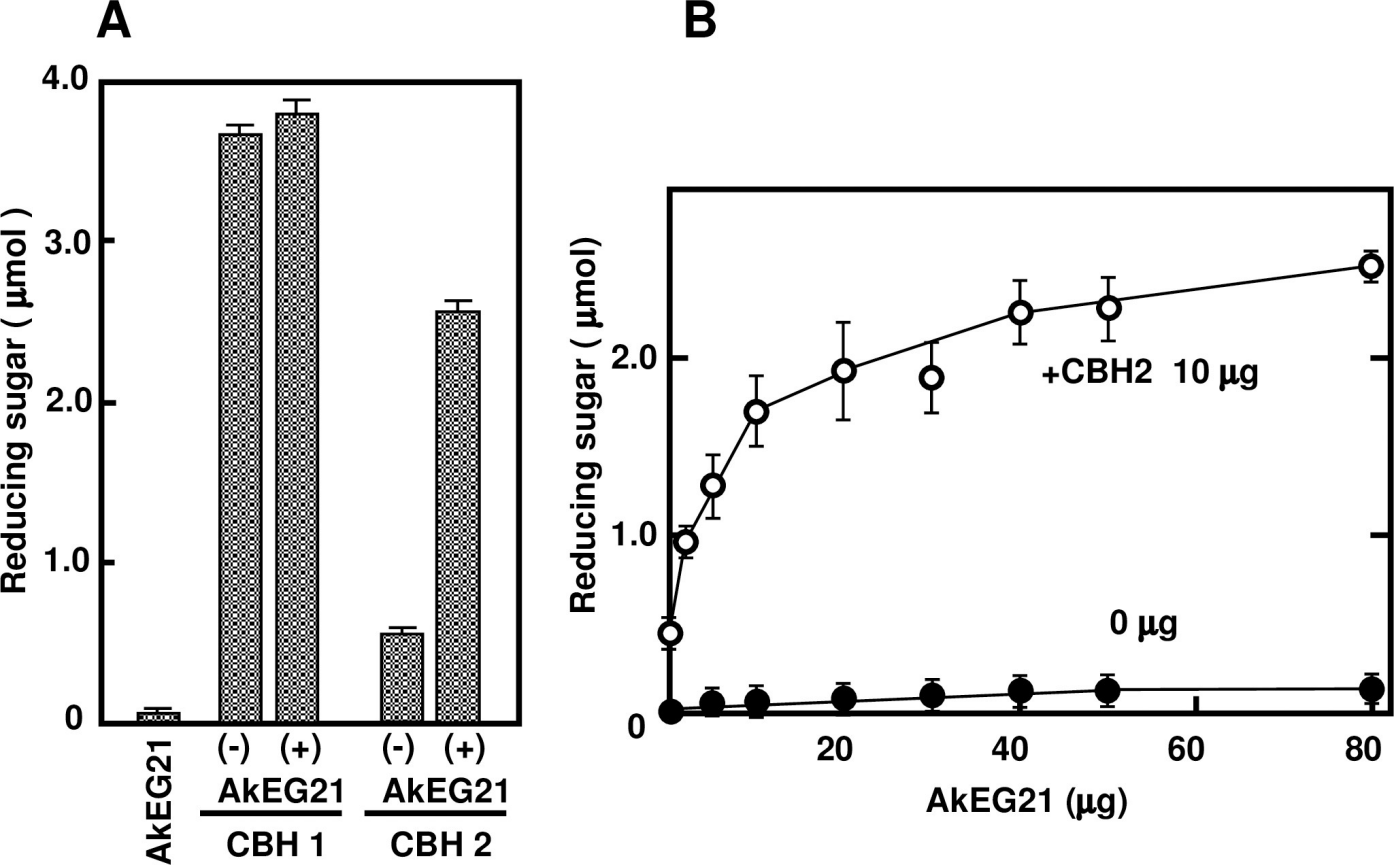
Hydrolysis of filter paper by synergistic activity of AkEG21 and CBH 2. (A) Filter paper was digested with CBH 1 or CBH 2 in the absence or presence of AkEG21 and AkEG21 alone at 37°C for 24 h. (B) Filter paper was digested with increasing amounts of AkEG21 in the absence or presence of CBH 2 at 37°C for 20 h. Cellulose hydrolysis was assessed by the release of reducing sugar. The data (mean ± standard deviation) were calculated from four separate experiments.

We next examined the synergy between AkEG21 and CBHs on cellulose hydrolysis by measuring reducing sugar as reaction products. When filter paper was incubated with AkEG21 and CBH 2, the release of reducing sugar was markedly increased compared to that in the reaction with AkEG21 or CBH 2 alone. The production of reducing sugar in the presence of AkEG21 and CBH 2 was about four-fold higher than the sum of the production by AkEG21 and CBH 2 alone. However, the addition of AkEG21 had no effect on cellulose hydrolysis by CBH 1 (Fig 10A). To confirm the synergistic effect of AkEG21 in the presence of CBH 2 on cellulose hydrolysis, filter paper was incubated with increasing concentration of AkEG21 in the presence of CBH2. Compared to the reaction with CBH 2 alone, cellulose hydrolysis was increased by AkEG21 in a concentration-dependent manner (Fig 10B). Thus a four-fold increase in cellulose hydrolysis was caused by the addition of AkEG21 to CBH 2 compared to CBH 2 alone. Further analysis is necessary to clarify the mechanism for the synergistic effect of AkEG21 and CBH 2 on cellulose hydrolysis.

### Effect of AkEG21 removal on the cellulolytic activity of *A*. *kurodai* digestive fluid

The digestive fluid of sea hare contains four ß-1,4-glucanases and two ß-glucosidases (110K and 210K BGL) as major cellulolytic components [22]. Although AkEG21 is the most abundant cellulase, its specific activity toward CMC is the lowest, constituting about 1/34 of that of AkEG45. Approximately 20 mg of purified AkEG21 could be obtained from 200 mL of the *A*. *kurodai* digestive fluid, i.e., the AkEG21 concentration in the digestive fluid is about 0.1 mg/mL. Although we showed synergistic effect of four cellulases and two ß-glucosidases on cellulose saccharification in seaweed [22], the contribution of AkEG21 to the cellulolytic activity of *A*. *kurodai* digestive fluid has not been examined.

**Fig 11:**
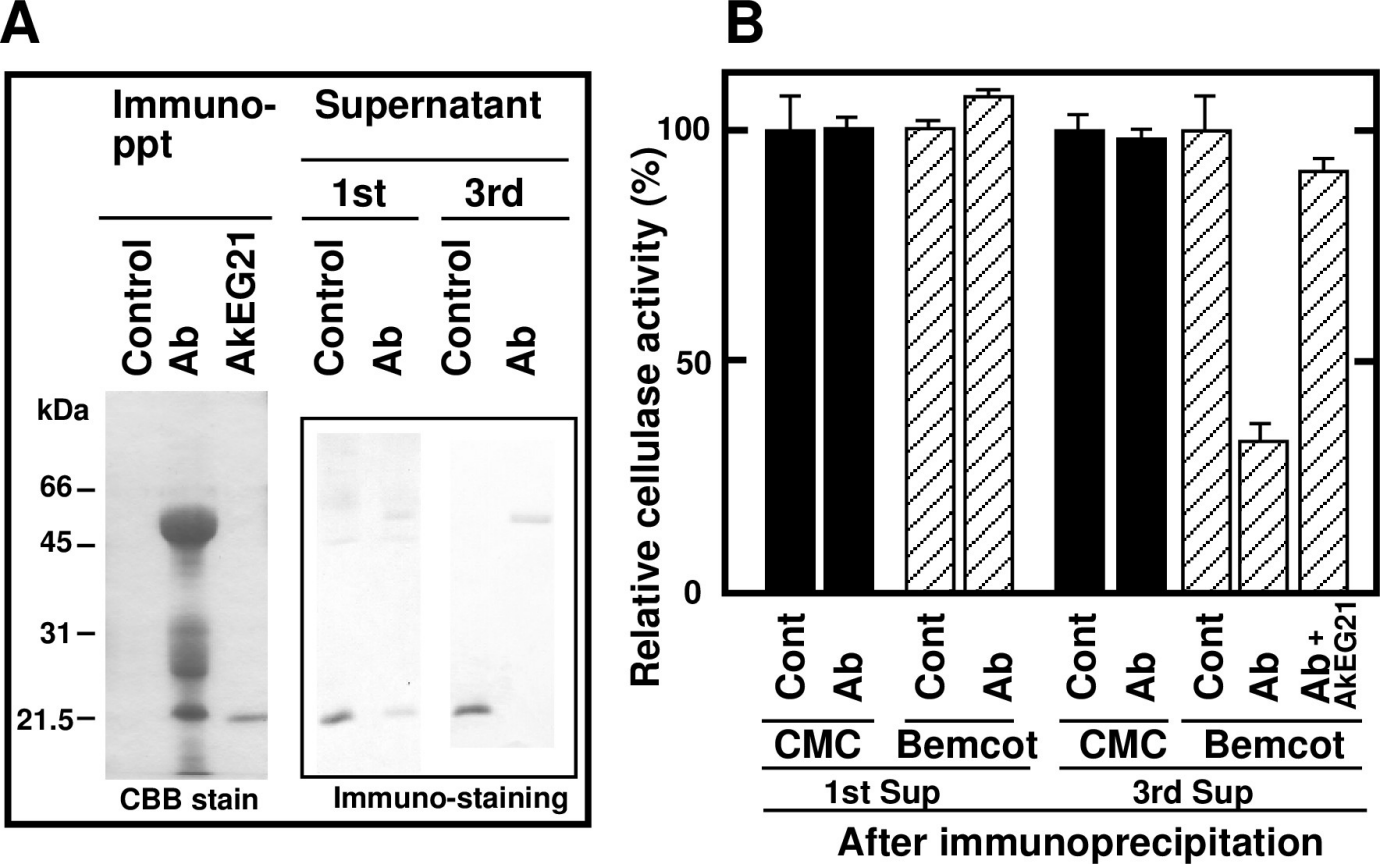
Effect of AkEG21 depletion on the hydrolytic activity of *A*. *kurodai* digestive fluid. (A) Removal of AkEG21 from the digestive fluid of sea hare using AkEG21-specific IgG was analyzed by SDS-PAGE followed by CBB staining or immunoblotting. (B) Bemcot and CMC hydrolysis by the digestive fluid after immunoprecipitation with anti-AkEG21 IgG. All values represent mean ± SD and each measurement was carried out in triplicate. Repeat experiment yielded similar results.

To clarify the role of AkEG21 in cellulose hydrolysis, AkEG21 was removed from the digestive fluid by immunoprecipitation with a specific anti-AkEG21 antibody (Fig 11A). A 21-kDa protein corresponding to AkEG21 was detected in the immunoprecipitate; however, complete removal of AkEG21 from the digestive fluid could be achieved only after the third round of immunoprecipitation (Fig 11A, right). The supernatant from the first precipitation retained the same activity toward CMC and Bemcot compared to the control digestive fluid as evidenced by the release of reducing sugar, although AkEG21 was greatly decreased in the supernatant (Fig 11B). As shown in Fig 9, trace amount of AkEG21 seemed to be enough to digest cellulose by synergistic action with other endoglucanases and exoglucanases. In contrast to first immunoprecipitation, the depletion of AkEG21 after the third immunoprecipitation resulted in a one-third reduction (32% of control) of digestive fluid hydrolytic activity toward Bemcot, which was restored by the addition of AkEG21 (Fig 11B). However, CMC hydrolysis was not affected by AkEG21 removal. As even a low concentration of AkEG21 exerted a synergistic effect on insoluble cellulose hydrolysis by AkEG45 (Fig 9), it is likely that trace amounts of AkEG21 remaining after the first immunoprecipitation still potentiated cellulolytic activity in *A*. *kurodai* digestive fluid. In contrast, CMC hydrolysis was performed efficiently without AkEG21 and, therefore, was not affected by AkEG21 removal. These results indicate that AkEG21 is an essential enzyme for the hydrolysis of insoluble cellulose by the digestive fluid of sea hare.

## Discussion

AkEG21 is the most abundant ß-1, 4-endoglucanase in the digestive fluid of *A*. *kurodai* as evidenced by its concentration (> 0.1 mg/mL), which is markedly higher than those of other 45-, 65- and 95-kDa cellulases. AkEG45 is the second major endoglucanase, but its concentration in the digestive fluid is considerably lower (5 μg/mL) compared to that of AkEG21. However, despite the high concentration, the specific activity of AkEG21 toward CMC (1.89 U/mg) is the lowest among cellulases in *A*. *kurodai* digestive fluid constituting about 2% of that of AkEG45. AkEG21 is an endo-type glycosidase as indicated by its effect on the viscosity of CMC solution [22], and sequence analysis revealed that the enzyme belongs to GHF45 cellulases lacking the CBD [23]. When AkEG21 was incubated with cellulose or cellohexaose, cellobiose was produced as a major product from both substrates, suggesting that AkEG21 possesses CBH activity in addition to endo-ß-1,4-glucanase activity [22]. Thus, we characterized the catalytic properties of AkEG21 and its synergy with other enzymes purified from *A*. *kurodai* digestive fluid and tried to clarify its role in the cellulose-degrading system of sea hare. However, the precise function of AkEG21 in seaweed cellulose digestion by *A*. *kurodai* is still unclear and the physiological significance of its unusual abundance and low enzymatic activity toward CMC remains uncertain.

Recently we identified a novel phlorotannin-binding protein, EHEP (Eisenia hydrolysis enhancing protein), which is indispensable for the efficient saccharification of *Eiseinia bicyclis* in the digestive fluid of sea hare by analyzing the difference between saccharification activity of the purified enzyme and crude digestive fluid [47]. Phlorotannins are present in brown seaweed, such as *E*. *bicyslis*, and strongly inhibit ß-glucosidase activity while EHEP protects ß-glucosidase from inhibition by phlorotannin. Although EHEP possesses three chitin binding domains, EHEP does not bind to chitin but binds to phlorotannins. Like EHEP, the role of AkEG21 in the digestive fluid may differ from predicted functions from its amino acid sequence. It is possible that AkEG21 plays a distinct role in the cellulose digestion system of sea hare which may be unrelated to its catalytic activity.

In this study, we aimed to further characterize the cellulolytic system of sea hare by identifying cellulose-binding proteins in the digestive fluid because cellulose-binding activity of cellulases and expansin-like proteins are critical for efficient cellulose digestion. The most remarkable finding of this study is that AkEG21, which lacks the CBD, can still bind to cellulose fibers of CF-11, Avicel, filter paper and Bemcot.

We verified our approach to screening of cellulose-binding proteins using Meicelase which is a complex of cellulases secreted from *T*. *viride*. Although Meicelase contains many cellulolytic enzymes and swollenin, only three cellulose-binding proteins (67, 57 and 37 kDa) were identified. Molecular masses of the cellulose binding proteins, 67-and 57-kDa protein are very similar to those of CBH 1 and 2, respectively and the N-termini of these proteins are blocked like CBH 1 and 2. Furthermore, the 67- and 57-kDa proteins possess strong Avicel binding activity and Avicel bound to 67 and 57 kDa proteins showed CBH activity. These results indicate that they are likely identical to CBH 1 and 2.

Cellulose-bound AkEG21 could not degrade an external substrate, azo-CMC (Fig 4C) but readily hydrolyzed internal cellulose substrates it was bound to; among them AkEG21 had the highest binding and catalytic preference to Bemcot, although the hydrolysis product profiles for different cellulose substrates at pH 5.5–6.5 were the same. Unlike typical endoglucanases which randomly cleave cellulose, AkEG21, in addition to its endoglucanase activity, could hydrolyze a variety of cellulose substrates with the release of cellobiose as a major reaction product. These results suggest that cellulose substrates with low crystallinity are preferred by AkEG21 over those with high crystallinity.

AkEG21 bound to cellulose showed synergy effects with AkEG45 in cellulose hydrolysis. The production of reducing sugar and total hexose from Bemcot and filter paper were markedly increased by the hydrolysis with the combination of AkEG21 and AkEG45 compared to that with AkEG21 or AkEG45 alone; however no synergy was observed in CMC hydrolysis. These results suggest that binding of AkEG21 to cellulose could promote the conversion of cellulose fibers network to a structure more susceptible to the cleavage by AkEG45, however, although AkEG21 can bind CMC, it may not change CMC structure.

The functional importance of AkEG21 for cellulose digestion by *A*. *kurodai* was confirmed by the effect caused by AkEG21 removal from the digestive fluid, which did not affect the hydrolysis of CMC but markedly reduced that of Bemcot (Fig 11), indicating an important role of AkEG21 in the entire cellulolytic system present in the digestive fluid of sea hare. These data are consistent with the notion that the mollusk β-1,4-endoglucanase belonging to GHF45, which is highly similar in sequence and enzymatic properties [23, 36–38], likely plays a critical role in the digestion of plant, seaweed and plankton cellulose.

CBHs are not common components of the cellulolytic system in microorganisms and it is thought that there are distinct microbial mechanisms involved in cellulose hydrolysis. Thus, CBH-independent pathways for cellobiose production from crystalline cellulose were detected in brown rot fungi, *Postia placenta* [12], *Gloeophyllum trabeum* [13] and marine bacterium, *Saccharophagus degradans* [15]. *Postia placenta* is an efficient cellulose-degrading organism, which, however, is lacking CBH-encoding genes [11,12]. It was reported that proccessive endoglucanases belonging to GHF5 play a significant role in cellulose hydrolysis instead of CBH [10–15]. Our present results strongly suggest that AkEG21 is a processive endoglucanase functionally equivalent to the CBH as it possesses both the endo- and exo-ß-1,4-glucanase activities. It is likely that after binding to cellulose fibers, AkEG21 first hydrolyzes ß-1,4 bonds of cellulose through endoglucanase activity and then cellulose to cellobiose, cellotriose, and cellotetraose through exoglucanase activity. As a result, AkEG21 binding to and hydrolysis of cellulose might convert the cellulose fibrous architecture to a structure more accessible to cleavage by other endoglucanases.

Further insights into the mechanism of AkEG21 catalytic activity can be provided by investigating the 3D conformation and structural determinants of its substrate-binding site. Structure analysis of several GHF45 cellulases from fungi indicated that they present a six-stranded ß-barrel [48–50]. A similar structure was also observed for GHF45 endoglucanase from blue mussel (PDB:1WC2), in which the substrate-binding channel is conformed mostly as a ß-barrel [50]. By mutating residues in the substrate-binding site predicted from the 3D-structure, the mode of AkEG21 activity could be clarified.

## Supporting information

S1 File Data underlying the study. (DOCX)
