# Supplementary material for: Cellulose-binding activity of a 21-kDa endo-ß-1,4-glucanase lacking cellulose-binding domain and its synergy with other cellulases in the digestive fluid of Aplysia kurodai
Source: PLoS One. 2018 Nov 9;13(11):e0205915. doi: 10.1371/journal.pone.0205915 (PMC6226162; doi:10.1371/journal.pone.0205915)
Supplement: S1 File — (DOCX) [file pone.0205915.s001.docx]

Fig. 2D（right） Effect of removal of 67- and 57-kDa proteins from Meicelase on glucose producing activity from filter paper of Meicelase.

|  | Glucose (mg) | Mean ± SD |
| --- | --- | --- |
| Control | 0.368  0.317  0.382  0.354 | 0.355 ± 0.03 |
| Avicel | 0.261  0.284  0.260 | 0.268 ± 0.015 |
| Bemcot | 0.055  0.049  0.048  0.044 | 0.053 ± 0.006 |

Fig. 4C Cellulase activity of filter paper-bound AkEG21 toward an external substrate azo-CMM.

|  | Azo-CMC cleaving activity | |
| --- | --- | --- |
|  | OD_590_ | Mean ± SD |
| Control | 0.371  0.317  0.375 | 0.354 ± 0.03 |
| Supernatant | 0.035  0.033  0.031 | 0.033 ± 0.002 |
| Filter paper | 0.035  0.02  0.02 | 0.025 ± 0.008 |

Fig. 7B The relative band intensity was calculated from four separate experiments using Image 3.

|  | Relative band intensity | | | |  | Relative Bemcot bound  AkEG21 band intensity (%) |
| --- | --- | --- | --- | --- | --- | --- |
|  | 1 | 2 | 3 | 4 | mean ± SD |  |
| Control | 0.95 | 0.90 | 0.809 | 0.70 | 0.839 ± 0.10 | 100 ± 11.9 |
| Glucose 5mg | 1.00 | 0.935 | 0.809 | 1.00 | 0.936 ± 0.09 | 112 ± 10.0 |
| Cellobiose 5mg | 0.746 | 0.780 | 1.00 | 0.840 | 0.842 ± 0.11 | 100 ± 13.0 |
| Maltose 5mg | 1.05 | 0.8 | 0.8 | 0.7 | 0.838 ± 0.15 | 99.9 ± 18.0 |
| Cellohexaose 1mg | 0.22 | 0.129 | 0.22 | 0.18 | 0.187 ± 0.04 | 22.2 ± 4.8 |
| S. starch 5mg | 0.90 | 1.00 | 0.940 | 0.80 | 0.910 ± 0.08 | 108 ± 9.5 |
| Laminaran 5mg | 0.800 | 0.806 | 0.72 | 0.777 | 0.776 ± 0.04 | 92.4 ± 5.0 |
| CMC 5mg | 0.42 | 0.41 | 0.41 | 0.42 | 0.415 ± 0.006 | 49.5 ± 0.70 |

Fig. 7C The relative band intensity was calculated from four separate experiments using Image 3.

|  | Relative band intensity | | | |  | Relative Bemcot bound  AkEG21 band intensity (%) |
| --- | --- | --- | --- | --- | --- | --- |
|  | 1 | 2 | 3 | 4 | mean ± SD |  |
| Control | 1.0 | 1.0 | 1.0 | 1.0 | 1.0 | 100 |
| Cellotriose | 0.40 | 0.28 | 0.24 | 0.27 | 0.298 ± 0.07 | 29.8 ± 7.0 |
| Cellopentaose | 0.25 | 0.22 | 0.193 | 0.18 | 0.210 ± 0.03 | 21.0 ± 3.0 |
| Cellohexaose | 0.186 | 0.08 | 0.09 | 0.143 | 0.1291 ± 0.05 | 12.9 ± 5.0 |

Fig. 9B Hydrolysis of Bemcot by the synergistic activity of AkEG21 and AkEG45. Bemcot was digested with increasing amount of AkEG21 in the absence or presence of AkEG45 at 37℃ for 20h. After reaction, reducing sugar liberated from Bemcot was determined.

|  | AKEG45 (10μg) | | | |
| --- | --- | --- | --- | --- |
|  | (−) |  | (＋) |  |
| AkEG21 |  | Reducing sugar (μmol) |  |  |
|  |  | (mean ± SD) |  | (mean ± SD) |
| 0 |  |  | ①　0.196  ②　0.138  ③　0.212 | 0.182 ± 0.038 |
| 10 | ①　0.023  ②　0.132  ③　0.004 | 0.073 ± 0.044 | ①　0.443  ②　0.368  ③　0.345 | 0.386 ± 0.04 |
| 25 | ①　0.581  ②　0.506  ③　0.891 | 0.659 ± 0.167 | ①　1.12  ②　1.32  ③　1.11 | 1.18 ± 0.096 |
| 50 | ①　0.558  ②　1.26  ③　2.11 | 1.31 ± 0.63 | ①　2.38  ②　3.31  ③　3.85 | 3.18 ± 0.61 |
| 75 | ①　1.72  ②　2.93  ③　2.90  ④　2.42 | 2.49 ± 0.49 | ①　3.20  ②　4.14  ③　4.72 | 4.73 ± 1.34 |
| 100 | ①　2.10  ②　2.77  ③　3.32 | 2.73 ± 0.50 | ①　6.08  ②　6.04  ③　6.07 | 6.06 ± 0.02 |
| 150 | ①　2.47  ②　3.29  ③　3.10  ④　3.28 | 3.04 ± 0.3 | ①　8.06  ②　6.83  ③　8.08  ④　8.00 | 7.74 ± 0.527 |
| 200 | ①　3.08  ②　3.61  ③　4.72  ④　4.81 | 3.98 ± 0.79 | ①　8.22  ②　8.83  ③　9.54  ④　8.69 | 8.82 ± 0.47 |

Fig. 9D Effect of AkEG45 on CMC digestion by AkEG21. CMC was digested with increasing amount of AkEG21 in the absence or presence of AkEG21 at 37℃ for 20 min and reducing sugar liberated from CMC was determined. The data were calculated from three separate experiments.

|  | AkEG45 (0.75μg) | | | | |
| --- | --- | --- | --- | --- | --- |
| AkEG21 | (−) | |  | (＋) | |
| (μg) | Reducing sugar (μmol) | |  | Reducing sugar (μmol) | |
|  |  | (mean ± SD) |  |  | (mean ± SD) |
| 0 |  |  |  | 0.274  0.250  0.250 | 0.258 ± 0.014 |
| 2 | 0.296  0.350  0.242 | 0.296 ± 0.044 |  | 0.547  0.649  0.781 | 0.659 ± 0.10 |
| 5 | 0.250  0.351  0.369 | 0.324 ± 0.05 |  | 0.826  0.948  0.632 | 0.802 ± 0.13 |
| 10 | 0.376  0.509  0.472 | 0.452 ± 0.06 |  | 1.02  1.06  0.902 | 0.994 ± 0.07 |
| 20 | 0.614  0.476  0.659 | 0.583 ± 0.08 |  | 0.826  1.15  0.870 | 0.947 ± 0.143 |
| 40 | 0.557  0.632  0.676 | 0.622 ± 0.05 |  | 1.04  1.13  1.08 | 1.08 ± 0.04 |
| 60 | 0.668  0.666 | 0.667 ± 0.001 |  | 0.966  0.973  0.966 | 1.07 ± 0.06 |
| 80 | 0.733  0.637  0.812 | 0.727 ± 0.07 |  | 1.06  0.973  0.966 | 1.00 ± 0.04 |

Fig. 10 Hydrolysis of filter paper by synergistic activity of AkEG21 and CBH2. (A) Filter paper was digested with CBH 1 or CBH 2 in the absence or presence of AkEG21 and AkEG21 alone at 37℃ for 24h.

|  | Reducing sugar (μmol) | |
| --- | --- | --- |
|  |  | (mean ± SD) |
| AkEG21　25μg | 0.115  0.079  0.077  0.152 | 0.105 ± 0.03 |
| CBH 1 | 3.55  3.60  3.50  3.67 | 3.58 ± 0.063 |
| CBH 1 ＋ AkEG21 | 3.90  3.96  3.94  3.71 | 3.88 ± 0.100 |
| CBH 2 | 0.544  0.600  0.755  0.586 | 0.621 ± 0.08 |
| CBH 2 ＋ AkEG21 | 2.70  2.55  2.40  2.56 | 2.55 ± 0.106 |

Fig. 10B Filter paper was digested with increasing amount of AkEG21 in the absence or presence of CBH2 at 37℃ for 20h.

|  | Reducing sugar (μmol) | | | |
| --- | --- | --- | --- | --- |
|  |  | CBH 2 (10μg) |  |  |
| AkEG21 | (−) |  | (＋) |  |
| (μg) |  | (mean ± SD) |  | (mean ± SD) |
| 0 | 0 | 0 | 0.261  0.555  0.394  0.522 | 0.433 ± 0.12 |
| 2 | 0  0  0 |  | 1.17  1.01  0.768 | 0.983 ± 0.165 |
| 5 | 0  0  0 |  | 1.24  1.58  1.13 | 1.32 ± 0.19 |
| 10 | 0.002  0.016  0.008 | 0.012 ± 0.004 | 1.50  1.38  1.98  1.82 | 1.07 ± 0.24 |
| 20 | 0.0172  0.0384  0.080 | 0.045 ± 0.026 | 1.36  1.90  2.09  2.25 | 1.90 ± 0.34 |
| 30 | 0.042  0.123  0.140 | 0.101 ± 0.04 | 1.43  1.84  1.96  2.13 | 1.84 ± 0.26 |
| 40 | 0.134  0.182 | 0.158 ± 0.024 | 2.00  2.53  2.22  2.38 | 2.28 ± 0.20 |
| 50 | 0.065  0.088  0.210 | 0.121 ± 0.063 | 2.0  2.43  2.19  2.55 | 2.29 ± 0.20 |
| 80 | 0.096  0.123  0.054 | 0.001 ± 0.03 | 2.50  2.60 | 2.55 ± 0.05 |

Fig.11 Effect of AkEG21 depletion on the hydrolytic (glucose producing) activity of A. Kurodai digestive fluid.

| Immuno precipitation | | CMC | | |  | Bemcot | | |
| --- | --- | --- | --- | --- | --- | --- | --- | --- |
|  |  | Glucose (mg/ml/h) | | % |  | Glucose (mg/ml/h) | | % |
|  |  |  | (mean ± SD) |  |  |  | (mean ± SD) |  |
| 1st | control | 12.7  14.6  13.9 | 13.7 ± 0.96 | 100 ± 7.0 |  | 0.290  0.298  0.296 | 0.295 ± 0.004 | 100 ± 1.4 |
|  | Anti-AkEG21 | 13.7  14.4  13.8 | 14.0 ± 0.37 | 102 ± 2.7 |  | 0.315  0.321  0.316 | 0.317 ± 0.013 | 107 ± 1.0 |
| 3rd | control | 14.3  15.8  15.1 | 15.1 ± 0.75 | 100 ± 4.9 |  | 0.228  0.248  0.214 | 0.230 ± 0.02 | 100 ± 8.6 |
|  | Anti-AkEG21 | 14.3  14.7  15.1 | 14.7 ± 0.4 | 97.3 ± 2.6 |  | 0.079  0.089  0.075 | 0.081 ± 0.01 | 35.2 ± 4.3 |
|  | Anti-AkEG21  AkEG21 | ND |  |  |  | 0.236  0.200  0.203 | 0.216 ± 0.01 | 93.6 ± 1.8 |
